# Identification of hub lncRNAs in head and neck cancer based on weighted gene co‐expression network analysis and experiments

**DOI:** 10.1002/2211-5463.13134

**Published:** 2021-05-21

**Authors:** Shao Lina

**Affiliations:** ^1^ Department of Endodontics School and Hospital of Stomatology China Medical University Shenyang China; ^2^ Liaoning Provincial Key Laboratory of Oral Diseases Shenyang China

**Keywords:** head and neck cancer, risk score prognostic model, The Cancer Genome Atlas, WGCNA

## Abstract

Head and neck squamous cell carcinoma (HNSCC) ranks as the sixth most common cancer among systemic malignant tumors, with 600 000 new cases occurring every year worldwide. Since HNSCC has high heterogeneity and complex pathogenesis, no effective prognostic indicator has yet been identified. Here, we aimed to identify a lncRNA signature associated with the prognosis of HNSCC as a potential new biomarker. LncRNA expression data were downloaded from The Cancer Genome Atlas database. A polygenic risk score model was constructed by using Lasso–Cox regression analysis. Weighted gene co‐expression network analysis (WGCNA) was applied to analyze the co‐expression modules of lncRNAs associated with the prognosis of HNSCC. The robustness of the signature was validated in testing and external cohorts. Polymerase chain reaction was performed to detect the expression levels of identified lncRNAs in cancer and adjacent tissues. We constructed an 8‐lncRNA signature (LINC00567, LINC00996, MTOR‐AS1, PRKG1‐AS1, RAB11B‐AS1, RPS6KA2‐AS1, SH3BP5‐AS1, ZNF451‐AS1) that could be used as an independent prognostic factor of HNSCC. The signature showed strong robustness and had stable prediction performance in different cohorts. WGCNA results showed that modules related to risk score mainly participated in biological processes such as blood vessel development, positive regulation of catabolic processes, and regulation of growth. The prognostic risk score model based on lncRNA for HNSCC may help clinicians conduct individualized treatment plans.

## Graphical abstract

I constructed an 8‐lncRNA signature (LINC00567, LINC00996, MTOR‐AS1, PRKG1‐AS1, RAB11B‐AS1, RPS6KA2‐AS1, SH3BP5‐AS1, ZNF451‐AS1) that may have potential as an independent prognostic factor of HNSCC. The signature showed strong robustness and good performance in different cohorts. My WGCNA results showed that modules related to risk score mainly participated in certain biological processes including blood vessel development and positive regulation of catabolic processes.

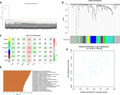

**Abbreviations**
HNSCC: head and neck squamous cell carcinoma
TCGA: The Cancer Genome Atlas
WGCNA: weighted gene co‐expression network analysis
GEO: Gene Expression Omnibus
OS: overall survival
GO: Gene Ontology
KEGG: Kyoto Encyclopedia of Genes and Genomes
ROC: receiver operative characteristic
GSEA: gene set enrichment analysis
AUC: area under the curve

Head and neck cancer ranks as the sixth most common cancer among systemic malignant tumors, with 600 000 new cases occurring every year worldwide [1]. Currently, the main treatments for head and neck cancer include surgery, chemotherapy, radiotherapy, and targeted therapy. Although these treatments are constantly being updated and progressing, the 5‐year overall survival (OS) rate of patients with head and neck squamous cell carcinoma (HNSCC) is about 50%, and this rate has not been improving [2]. Moreover, the 5‐year OS rate of HNSCC patients with distant metastasis is about 20%, indicating a serious threat to human life and health [3]. Therefore, it is greatly significant to predict the prognosis of HNSCC patients accurately in order to guide individualized treatment. Since HNSCC has high heterogeneity and complex pathogenesis, no effective prognostic indicator has yet been identified [4]. This represents an urgent need, specifically, new biomarkers to predict the long‐term survival rate of patients with HNSCC.

Long noncoding RNAs (lncRNAs) are RNA transcripts without protein‐coding ability. They are longer than 200 nucleotides, and they play an important role in regulating gene expression. LncRNAs began to attract the attention of academia since their function was discovered in 2007 [5]. Emerging evidence suggests that lncRNA may be involved in many diseases, and lncRNAs are expected to become new biomarkers for early diagnosis and prognosis prediction given their conservative secondary structures [6]. Many studies have shown that lncRNA expression is changed in gastric cancer, osteosarcoma, liver cancer, hepatoblastoma, pancreatic cancer, glioma, and other malignant tumors, suggesting that lncRNAs act as oncogenes or tumor suppressor genes in the processes of these malignant tumors [7, 8]. So far, there have been few studies on lncRNAs related to HNSCC, and most have been studies of single lncRNAs [9]. HOTAIR, MALAT1, lnc‐C22orf32‐1, lncTLR4‐1, lnc‐BCL2L11‐3, lnc‐AL355149.1‐1, and lnc‐ZNF674‐1 have been reported to play pivotal roles in HNSCC development and progression. However, the underlying molecular mechanisms are unclear [10, 11, 12]. Thus, further studies on the molecular mechanisms of lncRNAs in the development of HNSCC need to be conducted.

The Cancer Genome Atlas (TCGA) was created in 2006 in the United States, and it includes 20 000 patient samples and normal control samples as well as the clinicopathological features of 33 carcinomas, which are meant to accelerate the comprehensive study of human cancer gene mapping [13]. Tumor stage and grade in malignant phenotypes of HNSCC are closely related to the prognosis of HNSCC, so it is reasonable to identify prognostic lncRNAs by distinguishing different tumor subtypes of HNSCC [14].

In this study, the lncRNA expression profiles of HNSCC in the TCGA database were used to identify lncRNAs related to patient prognosis, and weighted gene co‐expression network analysis (WGCNA) was performed based on these lncRNAs to screen the tumor phenotype modules in order to identify the important biological processes involved. Finally, we identified lncRNAs related to survival by using multivariate Cox analysis, established a polygenic model that could accurately predict the prognostic risk of patients with HNSCC, and evaluated and validated the model to improve the clinical diagnosis and treatment of patients with HNSCC.

## Materials and methods

### Data acquisition and preprocessing

TCGA FPKM RNA sequencing data and the latest clinical follow‐up information were downloaded from the TCGA portal maintained by the Genomic Data Commons (https://gdc‐portal.nci.nih.gov/). The gene expression and prognostic data of the GSE41613 cohort were obtained from the Gene Expression Omnibus (GEO) database. We mapped the probe set IDs to the NetAffx annotation file to extract lncRNA expression data, and the probe set IDs were converted to Ensembl gene IDs. According to the annotation files, the probes were initially mapped into Ensembl annotation files (gencode.v28.long_noncoding_rnas.gtf) from the GENCODE website. Batch normalization was performed by the combat function in sva package, between the RNA‐seq data from the TCGA and the microarray data from the GEO database. The samples with no clinical information or OS < 30 days were removed, as were the normal tissue samples.

### Division of the training and testing data sets

A total of 499 samples from the TCGA database were divided into training and testing cohorts. To prevent deviation from affecting the stability of subsequent modeling, all samples were randomly assigned 100 times on the randomized in advance. The data were randomly partitioned, 50% into the training cohort and 50% into the independent testing cohort. The following conditions were used to choose the most suitable training cohort and testing cohort: distribution of age, clinical stage, follow‐up time, and death ratio. These conditions in the two groups were similar. The 97 samples of the GSE41613 cohort served as an external validation set.

### Screening of prognostic lncRNAs for head and neck squamous cell carcinoma

The survival package in R [15] was used to identify lncRNAs in the training cohort by univariate Cox regression. Genes with a *P*‐value < 0.05 were considered to be significantly related to OS. We further narrowed the gene range and built a prognostic model while maintaining high accuracy. The glmnet package in R [16] was used to perform Lasso–Cox regression analysis. The Lasso method is a compressed estimation. It results in a more refined model by constructing a penalty function, compressing some coefficients, and setting others to 0. It therefore retains the advantages of subset shrinkage. It is a biased estimation for processing data with multicollinearity. It can realize the selection of variables while estimating parameters and solve the problem of multicollinearity that is present in regression analysis.

Multivariate Cox regression analysis was then performed to determine the genetic risk characteristics and their corresponding coefficients. The risk score of each patient was calculated by multiplying the expression value of the gene by the corresponding coefficient. Next, patients were divided into high‐ and low‐risk groups according to the median risk score. We used the timeROC package for prognostic classification of the risk score, and we analyzed the classification efficiency of OS prediction for 3 and 5 years. The difference in OS between the high‐ and low‐risk groups was analyzed by using the Kaplan–Meier method.

### Weighted gene co‐expression network analysis of risk score modules

We obtained 168 lncRNAs associated with prognosis (*P* < 0.05) according to the results of the univariate Cox analysis. To identify the co‐expression modules of lncRNAs related to HNSCC prognosis and biomarkers related to risk score, we built a weighted co‐expression network using the WGCNA package in R [17]. The metabolic network is a typical sort of scale‐free network; in other words, there is a significant negative correlation between the logarithm of the connection degree of the node log (k) and the logarithm of the probability of the node log (P (k)), and the correlation coefficient is > 0.8. Thus, we chose β equal to 6 to ensure that the network was scale‐free.

Next, we converted the expression matrix into the adjacency matrix and then converted the adjacency matrix into the topology matrix. We used the business‐linkage hierarchical clustering method to cluster genes based on the topological overlap measure by using the Dynamic Tree Cut method, and the minimum number of lncRNAs in each network module was 5. After identifying modules by the Dynamic Tree Cut method, we calculated an eigenvector for each module, then performed cluster analysis on the modules. All the closed modules were merged into a new module. To calculate the correlations between the genes and clinical information, conditions were set as follows: height = 0.25, deepSplit = 2, and minModuleSize = 5. Finally, we analyzed the significant correlations between the modules and HNSCC.

We used miRcode [18] (http://www.mircode.org) to determine the interactions between lncRNA and miRNA. Then, we searched for the target gene of the miRNA by using the miRDB [19], miRTarBase [20], and TargetScan [21] databases. After the lncRNA‐miRNA and miRNA–mRNA pairs were determined, Cytoscape v3.7 software was used to build the DEmRNA‐DElncRNA‐DEmiRNA network. The mRNAs in the ceRNA network directly performed the biological functions, so we carried out Gene Ontology (GO) and Kyoto Encyclopedia of Genes and Genomes (KEGG) enrichment analyses to understand the biological functions of the network.

### Relationships between risk score and clinical characteristics

Univariate and multivariate Cox regression analyses in both the training and validation sets were performed to determine whether the risk score and clinicopathological features were independent factors of OS in patients with HNSCC. The clinicopathological features were considered independent OS features when the *P*‐value was < 0.05.

To determine whether the risk score obtained by the model was correlated with clinical characteristics, categorical variables were grouped according to clinical characteristics. We removed samples with incomplete clinical information and found whether the risk scores of the two groups were significantly different by using t‐tests. The risk scores of the different groups were significantly different when the *P*‐value was < 0.05.

### Gene set enrichment analysis

GSEA 4.0.3 software [22] (http://software.broadinstitute.org/gsea/index.jsp) was used for the gene set enrichment analysis (GSEA). All the samples were divided into high‐ and low‐risk groups by using the critical value of the training cohort. GSEA was utilized to identify the potential functions of the lncRNAs. The annotated gene set ‘c2.cp.kegg.v7.0.symbols.gmt’ was selected as the reference gene set. A false discovery rate < 0.05 was considered significant.

### External validation

We verified the accuracy of the 8‐lncRNA signature based on the external validation set, and we divided the samples into high‐ and low‐risk groups by using the median value. The receiver operative characteristic (ROC) curve was used to further evaluate the predictive power of the model, and Kaplan–Meier analysis was used to assess the OS between the high‐ and low‐risk samples determined by the risk score model.

### Quantitative reverse transcription‐polymerase chain reaction validation of lncRNA expression

Twenty pairs of HNSCC and tumor‐adjacent normal tissues collected from the Department of Endodontics, School and Hospital of Stomatology, China Medical University, were included for validation. The experiments were undertaken with the understanding and written consent of each subject. The study methodologies conformed to the standards set by the Declaration of Helsinki and were approved by the China Medical University ethics committee.

Total RNA was extracted by using TRIzol Reagent (Invitrogen, Carlsbad, CA, USA) following the manufacturer's protocol, and it was reverse‐transcribed into cDNA by using a Superscript Reverse Transcriptase Kit (Transgene, Strasbourg, France). A Super SYBR Green Kit (Transgene) was used to perform real‐time polymerase chain reaction (PCR) in an ABI7300 Real‐Time PCR System (Applied Biosystems, Foster City, CA, USA). The *GAPDH* gene was used as an internal reference, and the experiments were repeated in triplicate. The primer pairs were as follows: LINC00567 forward: ATCTGCCCTCCAGTGGATCT, LINC00567 reverse: AGGGGCTTTCCCCATTTAGC;

LINC00996 forward: TGGTAGGTCGGGGTAGTCA, LINC00996 reverse: ACAGTCTCCTTGGGGCATTG;

MTOR‐AS1 forward: TCCCATCTTTTCTGCCGGTC, MTOR‐AS1 reverse: GAAATGCTCCCCTCAACCCA;

PRKG1‐AS1 forward: ATCTTAGCAGTTGGCAGCGT, PRKG1‐AS1 reverse: GAGCTCTCCACGACGTCAAA;

RAB11B‐AS1 forward: AACCGTACCTTGAAAGCCCC, RAB11B‐AS1 reverse: AGGCTTCTAATACTTTTTGGACTTG;

RPS6KA2‐AS1 forward: CAAGTCCAAAAAGTATTAGAAGCC, RPS6KA2‐AS1 reverse: TGGAAGAAAATGTTTGCAAGAAGGA;

SH3BP5‐AS1 forward: CAAGTCCAAAAAGTATTAGAAGCCT, SH3BP5‐AS1 reverse: TGGTGTCATGTACAGATTTGGAT;

ZNF451‐AS1 forward: ACCGAAGAGGCAGTTATGGC, ZNF451‐AS1 reverse: GCAAATTCTTACTGAACTCATGTTG; and

GAPDH forward: ACCCAGAAGACTGTGGAGG, GAPDH reverse: TTCTAGACGGCAGGTCAGGT.

## Results

### Flowchart

**Fig. 1:**
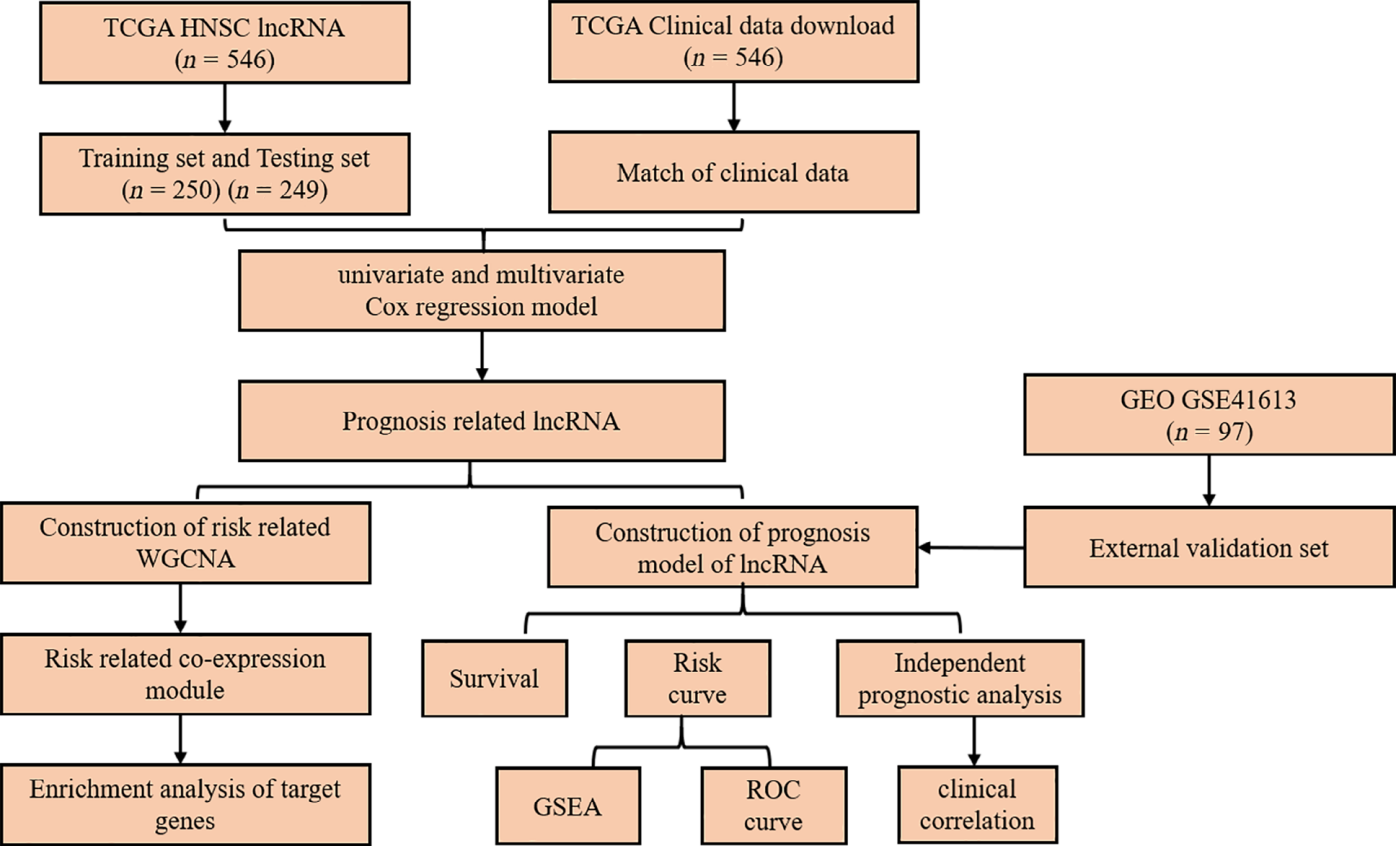
Flowchart of the method of this study.

To better understand the research idea of this paper, we drew a flowchart (Fig. 1).

### Data preprocessing

We obtained 549 RNA sequencing samples from the TCGA database. A total of 13 689 lncRNA transcripts from 499 preprocessed samples with follow‐up information were selected for further study. Samples were divided into two groups according to a training cohort‐to‐testing cohort ratio of 1 : 1 by random sampling. The final training cohort consisted of 250 samples, while the final testing cohort consisted of 249 samples. The GSE41613 cohort contained 97 samples. The clinical information statistics of the three cohorts after pretreatment are shown in Table 1.

**Table 1:** Clinical information statistics of three cohorts after preprocessing.

Characteristic		TCGA training cohort	TCGA testing cohort	GSE41613
Survival status	Alive	141	141	46
	Dead	109	108	51
Stage	I/II	43	51	41
	III/IV	171	166	56
Age	< 60	102	118	50
	>= 60	148	131	47
Sex	F	74	59	31
	M	176	190	66
Grade	G1	43	18	–
	G2	133	165	–
	G3	64	55	–
	G4	0	2	–
	GX	9	7	–
T	T0	0	1	–
	T1	24	21	–
	T2	59	72	–
	T3	45	51	–
	T4	92	79	–
	TX	17	16	–
N	N0	87	83	–
	N1	33	32	–
	N2	71	93	–
	N3	4	3	–
	NX	40	29	–
M	M0	95	90	–
	M1	0	1	–
	MX	29	32	–
Total	–	250	249	97

### Construction of the prognostic 8‐lncRNA model

**Fig. 2:**
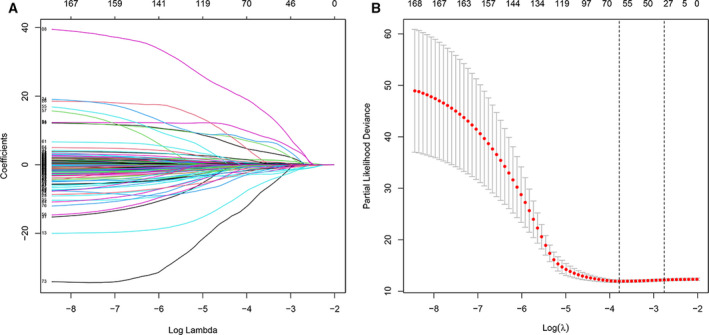
(A) The Lasso regression model and cross‐validation method were used to screen lncRNAs. When the number of variables was 59, we obtained the minimum partial likelihood deviance. (B) Regression coefficient graph of lncRNAs in the Lasso regression model.

Univariate Cox analysis was performed to screen lncRNAs related to prognosis based on the 250 samples in the training cohort. There were 168 lncRNAs with a significant difference in OS (log‐rank *P* < 0.05). The large number of lncRNAs was not conducive to clinical detection, so we further narrowed the range while maintaining high accuracy. We used the Lasso regression to compress the 168 prognostic lncRNAs. First, we analyzed the trajectory of each independent variable change (Fig. 2A). The lambda increased gradually, and the number of independent variable coefficients tending to 0 also increased gradually. We used threefold cross‐validation for model construction. Through the analysis of each lambda confidence interval (Fig. 2B), we found that the model achieved the optimum with a lambda value of 0.000251, so 59 genes at lambda = 0.000251 were selected as target genes.

The coefficients generated by multivariate Cox analysis were used to calculate the risk score of each patient using the following formula: risk score = gene expression value multiplied by the corresponding coefficient in summation. Finally, 8 lncRNA risk score models were obtained (Table 2), and the 8‐mRNA signature formula was as follows: RiskScore = −3.16*exp^LINC00567^−2.807*exp^LINC00996^−14.543*exp^MTOR‐AS1^+5.184*exp^PRKG1‐AS1^−0.212*exp^RAB11B‐AS1^−24.845*exp^RPS6KA2‐AS1^+0.864*exp^SH3BP5‐AS1^−6.759*exp^ZNF451‐AS1^.

**Table 2:** 8‐lncRNA multivariate cox analysis.

id	coef	HR	HR.95L	HR.95H	*P*‐value
LINC00567	−3.16	0.907	0.831	0.990	0.029
LINC00996	−2.807	0.865	0.721	1.038	0.118
MTOR‐AS1	−14.543	0.665	0.321	1.377	0.272
PRKG1‐AS1	5.184	1.784	0.858	3.708	0.001
RAB11B‐AS1	−0.212	0.809	0.669	0.978	0.029
RPS6KA2‐AS1	−24.845	0.32	0.125	0.820	0.018
SH3BP5‐AS1	0.864	2.372	1.156	4.866	0.019
ZNF451‐AS1	−6.759	0.623	0.420	0.922	0.018

### Assessment of the prognostic 8‐lncRNA model

**Fig. 3:**
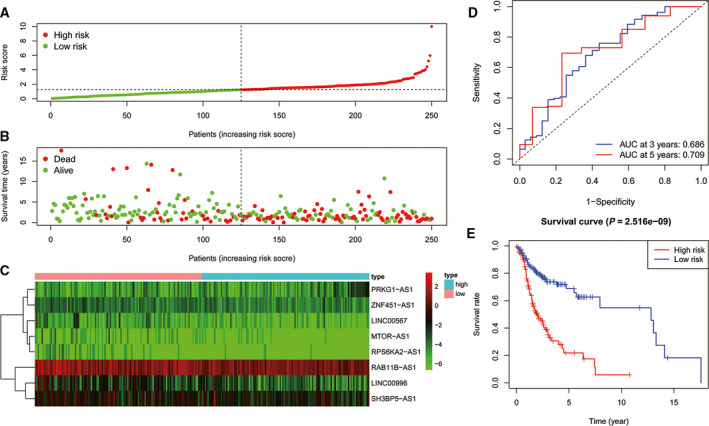
(A) Distribution of risk scores of patients with HNSCC in the training cohort. (B) Risk scores and survival states of patients with HNSCC in the training cohort. (C) Heat map of risk scores based on lncRNA expression in patients with HNSCC in the training cohort. (D) ROC curve of the prognostic model constructed in the training cohort. (E) Kaplan–Meier survival curve of high‐ and low‐risk patients’ OS rates in the training cohort.

To evaluate the effect of the model on HNSCC prognosis, patients in the training cohort were divided into high‐ and low‐risk groups according to the median risk score value. Fig. 3A–C shows the distribution of risk scores based on the 8‐lncRNA signature in the training cohort. Kaplan–Meier analysis results showed that the OS in the high‐risk group was significantly lower than that in the low‐risk group (*P* < 0.001, Fig. 3E).

We performed ROC analysis on the risk score for prognostic classification by using the timeROC package at 3 and 5 years in the training cohort (Fig. 3D). The area under the curve (AUC) for 3 years was 0.686, and for 5 years, it was 0.709.

**Fig. 4:**
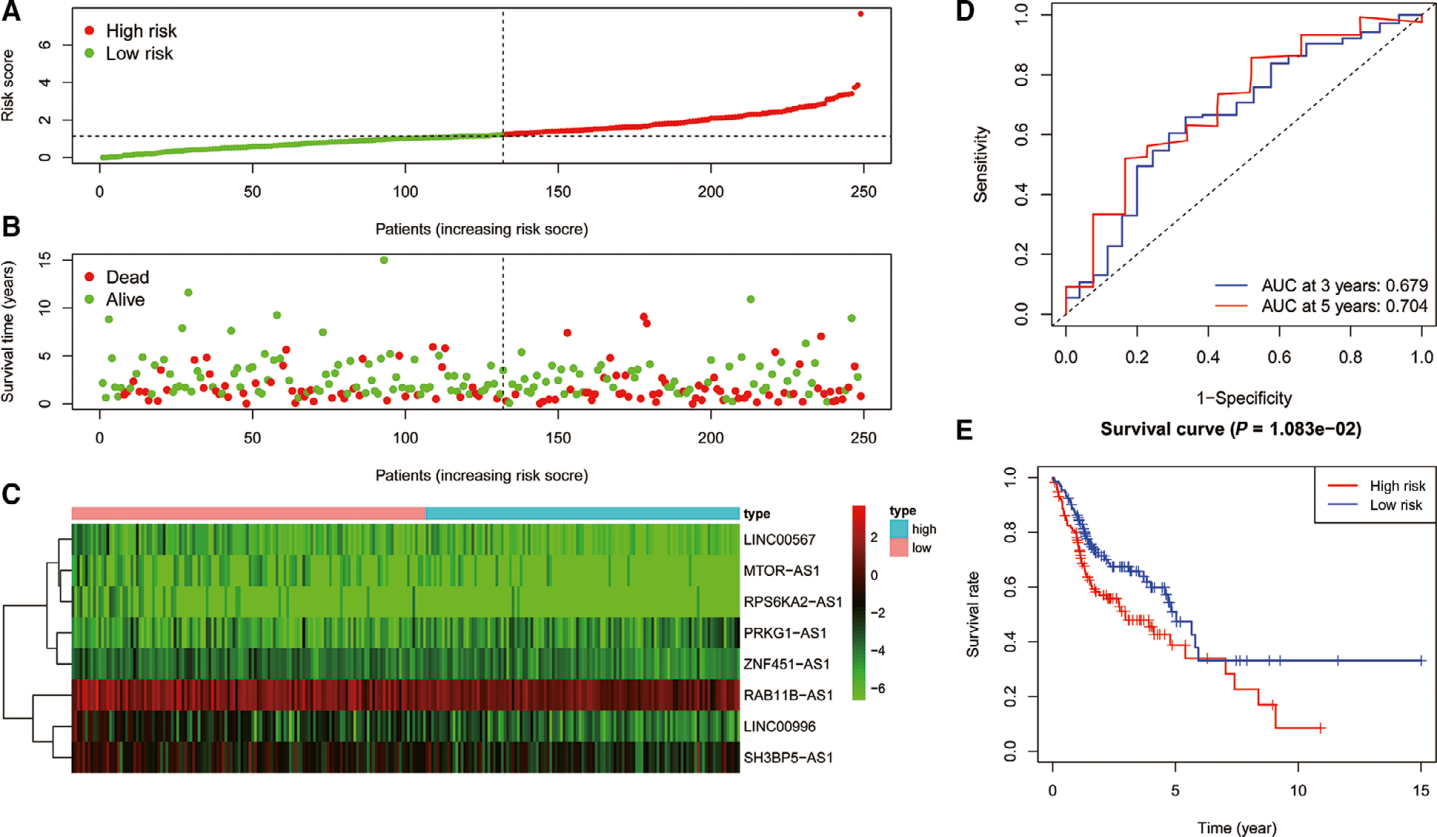
(A) Distribution of risk scores of patients with HNSCC in the testing cohort. (B) Risk scores and survival states of patients with HNSCC in the testing cohort. (C) Heat map of risk scores based on lncRNA expression in patients with HNSCC in the testing cohort. (D) ROC curve of the prognostic model constructed in the testing cohort. (E) Kaplan–Meier survival curve of high‐ and low‐risk patients’ OS rates in the testing cohort.

Similar results were obtained in the testing cohort; the AUC for 3 years was 0.679, and for 5 years, it was 0.704. Kaplan–Meier analysis results showed that the OS in the high‐risk group was significantly lower than that in the low‐risk group (*P* = 0.011, Fig. 4A–E).

**Fig. 5:**
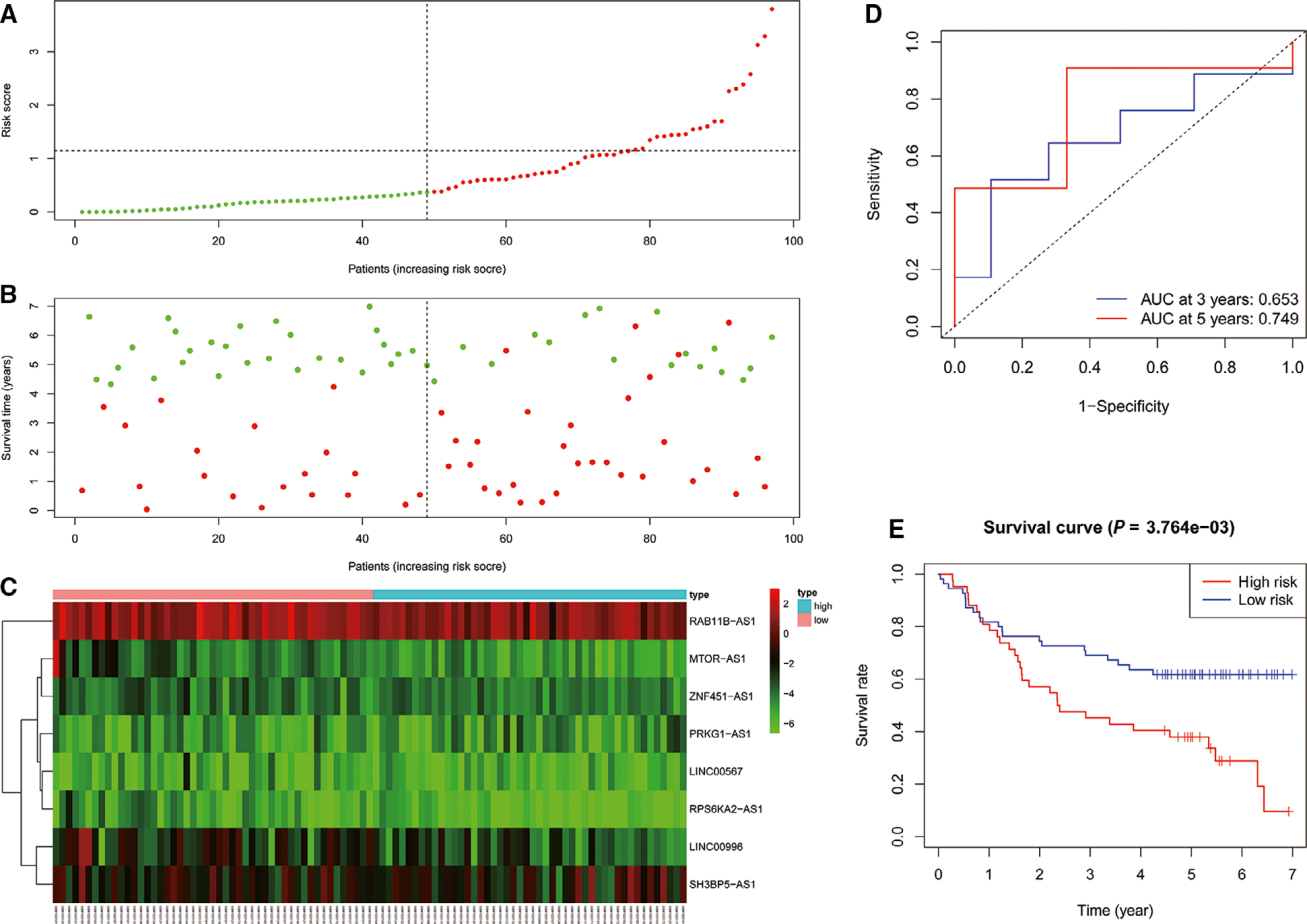
(A) Distribution of risk scores of patients with HNSCC in the external validation cohort. (B) Risk scores and survival states of patients with HNSCC in the external validation cohort. (C) Heat map of risk scores based on lncRNA expression in patients with HNSCC in the external validation cohort. (D) ROC curve of the prognostic model constructed in the external validation cohort. (E) Kaplan–Meier survival curve of high‐ and low‐risk patients’ OS rates in the external validation cohort.

We used the same method to calculate the lncRNA risk signature in the GSE41613 cohort. The results showed that the 3‐year AUC was 0.653, and the 5‐year AUC was 0.749. The OS in the high‐risk group was significantly worse than that in the low‐risk group (*P* = 0.0038, Fig. 5A–E) according to the median value. Our results suggested that the 8‐lncRNA risk model could effectively distinguish the OS of patients with HNSCC in different cohorts.

To prove the robustness of the signature, we included the GSE41613 cohort, which was preprocessed according to Data acquisition and preprocessing. We applied the same model and coefficients as the training cohort to the GSE41613 validation cohort and analyzed the ROCs of the samples’ risk scores. The results showed that the 3‐year AUC was 0.653, and the 5‐year AUC was 0.749.

**Fig. 6:**
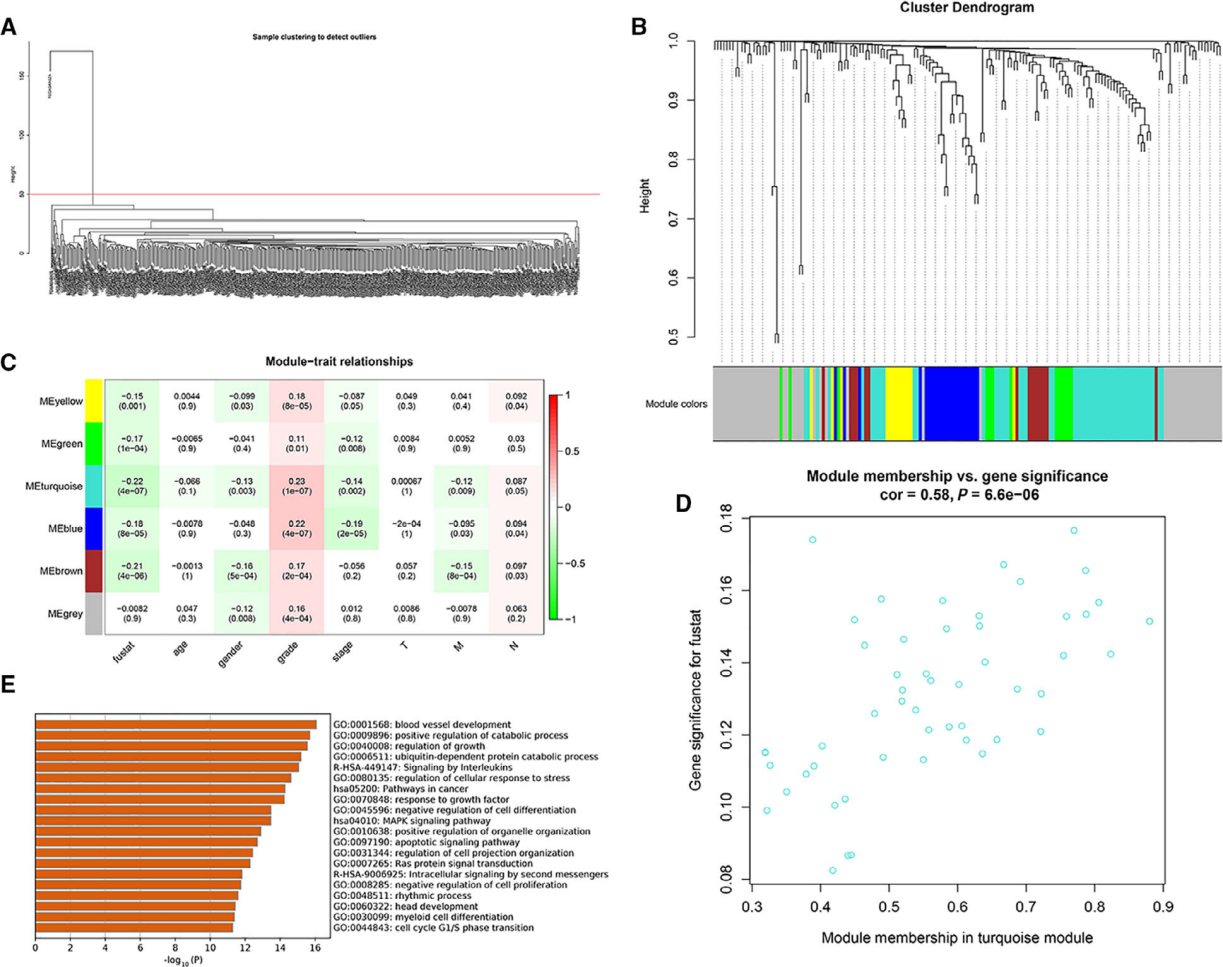
(A) Hierarchical cluster analysis to remove outliers. (B) Gene clustering dendrogram according to the adjacency‐based dissimilarity of hierarchical clustering. The color piece below represents the module identified by the Dynamic Cut Tree method. (C) Heat map of correlations between the module and clinical characteristics. The number represents the correlation in the color piece, and the *P*‐value is below. Red is positively correlated, and green is negatively correlated. (D) Chart of the results of the GO and KEGG enrichment analyses of the turquoise module. The length of the bar represents the number of genes enriched, and the names on the right are the pathway names. (E) Scatter diagram of the correlations between the turquoise module genes and fustat.

We conducted *z*‐score transformation of the risk scores, dividing the samples with risk scores > 0 into the high‐risk group and those with risk scores < 0 into the low‐risk group (Fig. 6B,D).

### Weighted gene co‐expression network analysis of risk score‐related modules

Hierarchical clustering analysis was carried out on the samples, and outliers were eliminated before WGCNA (Fig. 6A). Based on the Dynamic Cut Tree algorithm, 6 gene modules were obtained (Fig. 6B), and the gray module included all the genes that could not be clustered. We calculated the correlations between the 6 gene modules and age, sex, stage, risk, and other clinical information (Fig. 6C). We found that the blue, turquoise, yellow, green, and brown modules were negatively correlated with fustat and positively correlated with grade, while the blue and turquoise modules were negatively correlated with stage.

The blue, turquoise, yellow, green, and brown modules contained 20, 42, 7, 9, and 9 lncRNAs, respectively. The relationships between the rest of the modules and clinical features were weakly relevant or irrelevant. The module and clinical feature with the highest correlation were turquoise module and fustat. As shown in Fig. 6D, the absolute correlation coefficient between the turquoise module and fustat was Pearson Cor = 0.58, with this module showing the highest correlation with HNSCC, and the correlation was significant (*P* < 0.001), so it was selected as the hub module. The target genes regulated by the lncRNAs in the hub module are shown in Table S1. The enrichment analysis results are shown in Fig. 6E. The module was mainly involved in the biological processes of blood vessel development, positive regulation of catabolic processes, regulation of growth, ubiquitin‐dependent protein catabolic processes, signaling by interleukins, regulation of cellular response to stress, pathways in cancer, response to growth factor, negative regulation of cell differentiation, and the MAPK signaling pathway.

### LncRNA‐based risk score is an independent feature of overall survival for head and neck squamous cell carcinoma

To determine whether risk scores could be used as independent OS indicators, univariate and multivariate Cox regression analyses were performed in the training cohort. Univariate Cox analysis results showed that the 8‐lncRNA risk score was significantly associated with worse prognosis, with a hazard ratio (HR) of 1.700 (*P* < 0.001, 95% CI: 1.284–2.251, Table 3). Moreover, grade (HR = 1.759, 95% CI: 1.068–2.898, *P* = 0.027) and N stage (HR = 1.506, 95% CI: 1.054–2.152, *P* = 0.025) were also significantly correlated with OS. We then included all variables in the multivariate Cox analysis. The 8‐lncRNA risk score remained a risk factor for worse OS in patients with HNSCC (HR = 1.794, 95% CI: 1.255–2.565, *P* = 0.001). Thus, it was suggested that the 8‐lncRNA signature was an independent OS factor for HNSCC.

**Table 3:** Univariate and multivariate cox analyses of 8‐gene signature in training cohort.

Variables	Univariable analysis				Multivariable analysis			
	HR	95% CI of HR		*P*	HR	95% CI of HR		*P*
		Lower	Upper			Lower	Upper	
Age	1.005	0.978	1.033	0.697	0.996	0.964	1.029	0.817
Gender	0.744	0.376	1.472	0.396	0.515	0.248	1.070	0.075
Grade	1.759	1.068	2.898	0.027	1.489	0.848	2.614	0.166
Stage	1.577	0.969	2.568	0.067	0.809	0.361	1.814	0.607
T	1.295	0.917	1.830	0.142	1.342	0.769	2.342	0.300
M	0.726	0.254	2.071	0.549	0.462	0.136	1.572	0.216
N	1.506	1.054	2.152	0.025	1.433	0.928	2.215	0.105
RiskScore	1.700	1.284	2.251	0.000	1.794	1.255	2.565	0.001

### Gene set enrichment analysis

**Fig. 7:**
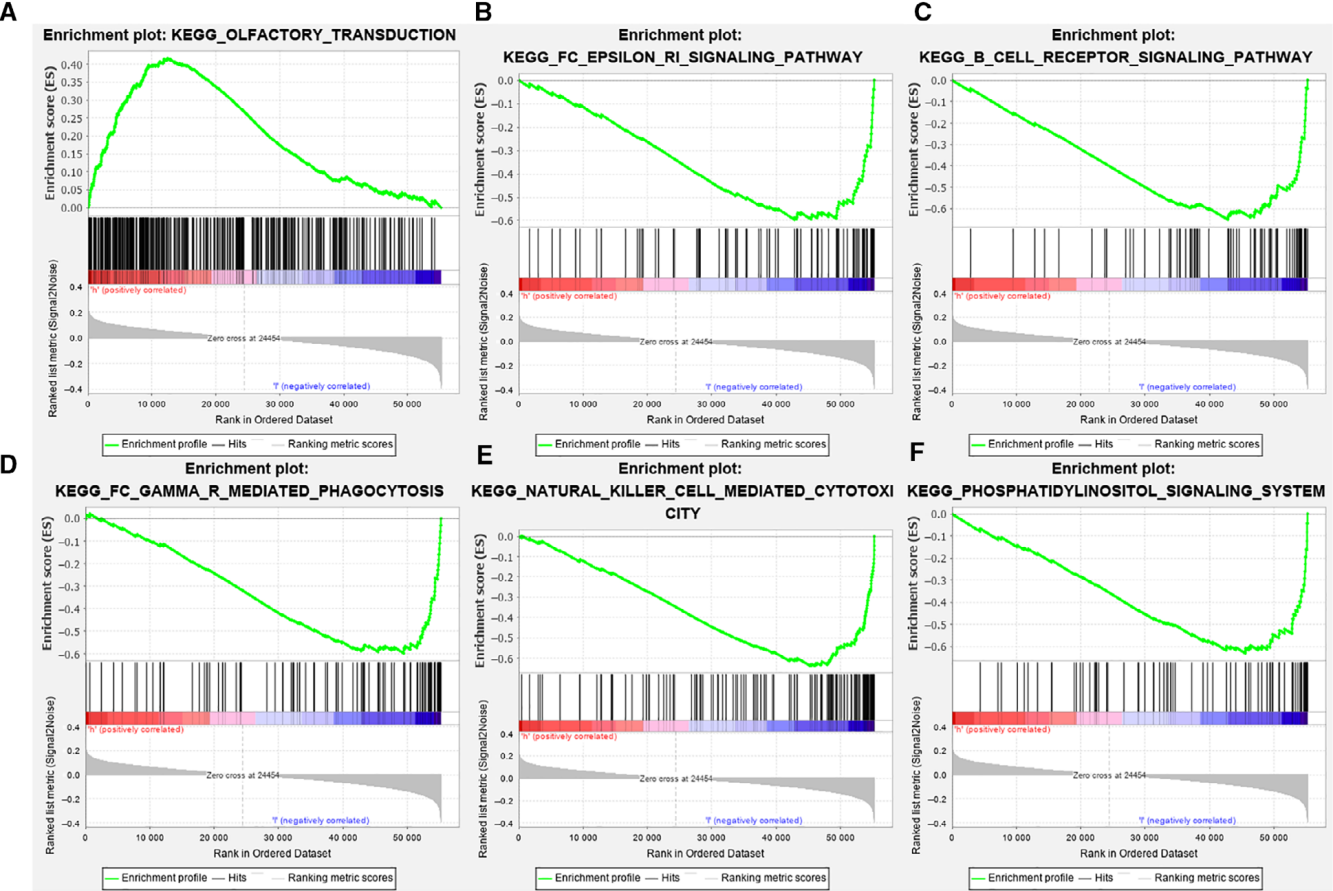
GSEA results based on the training cohort samples.

GSEA results indicated that in the training cohort, the high‐risk group was mainly enriched in OLFACTORY_TRANSDUCTION, while the low‐risk group was mainly enriched in NATURAL_KILLER_CELL_MEDIATED_CYTOTOXICITY, PHOSPHATIDYLINOSITOL_SIGNALING_SYSTEM, FC_GAMMA_R_MEDIATED_PHAGOCYTOSIS, FC_EPSILON_RI_SIGNALING_PATHWAY, B_CELL_RECEPTOR_SIGNALING_PATHWAY, PRIMARY_IMMUNODEFICIENCY, T_CELL_RECEPTOR_SIGNALING_PATHWAY, CHEMOKINE_SIGNALING_PATHWAY, NON_SMALL_CELL_LUNG_CANCER, VASCULAR_SMOOTH_MUSCLE_CONTRACTION, CELL_ADHESION_MOLECULES_CAMS, ANTIGEN_PROCESSING_AND_PRESENTATION, and ACUTE_MYELOID_LEUKEMIA (Fig. 7A–F).

### Quantitative reverse transcription‐polymerase chain reaction validation of the expression levels of the 8 lncRNAs

**Fig. 8:**
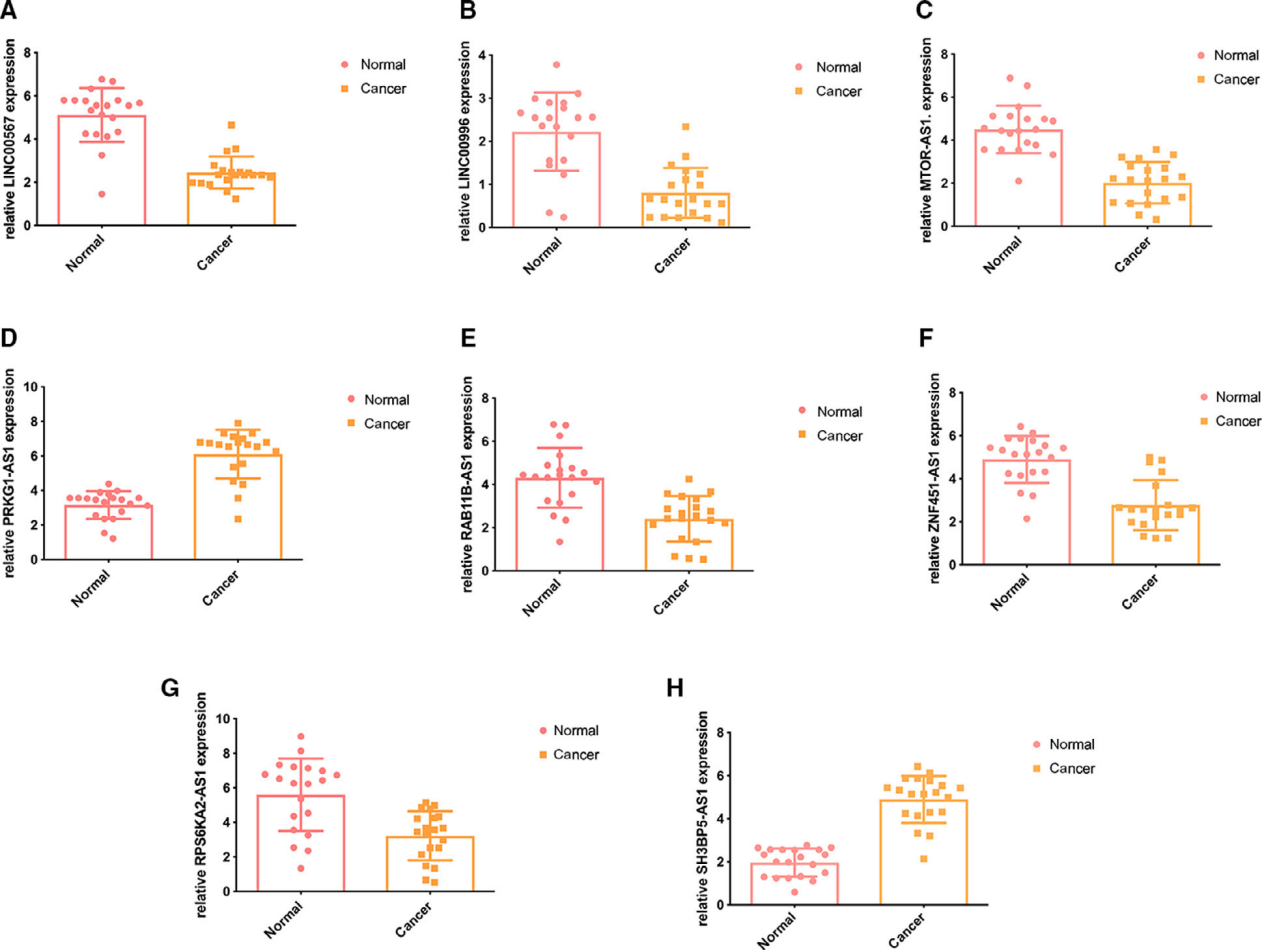
PCR of the eight lncRNAs in the HNSCC and normal samples. (A) Expression of LINC00567. (B) Expression of LINC00996. (C) Expression of MTOR‐AS1. (D) Expression of PRKG1‐AS1. (E) Expression of RAB11B‐AS1. (F) Expression of ZNF451‐AS1. (G) Expression of RPS6KA2‐AS1. (H) Expression of SH3BP5‐AS1. Error bars represent means ± SD.

The results of quantitative reverse transcription‐PCR showed that PRKG1‐AS1 and SH3BP5‐AS1 were significantly upregulated in HNSCC samples compared with normal samples. In addition, LINC00567, LINC00996, MTOR‐AS1, RAB11B‐AS1, RPS6KA2‐AS1, and ZNF451‐AS1 were significantly downregulated in tumor samples compared with normal samples (Fig. 8).

## Discussion

Adverse prognostic factors, such as tumor stage, tumor grade, tumor size, lymph node metastasis, and chemotherapy drug resistance, are considered to be closely related to HNSCC risk [23]. In addition, mRNAs and miRNAs as biomarkers to predict the risk of HNSCC recurrence have been widely applied in studies [24, 25]. However, there are few studies of lncRNAs as prognostic biomarkers for HNSCC, and the biological mechanisms of recurrence are unclear.

Traditional studies usually focus on the effect of a certain lncRNA on cancer. Because the occurrence and development of cancer are very complex, with the involvement of multiple genes and abnormal signaling pathways, studying the effect of a single gene on cancer has limitations. At present, many data make it possible to understand and study tumors at the genome level. The establishment of the TCGA database, the GEO database, and other large cancer databases has enabled researchers to obtain large gene expression profiles. Therefore, with the help of several algorithms, we established a risk score to quantify the relationships between lncRNAs and prognosis in HNSCC, and we clarified the interactions between prognosis, clinical features, and lncRNAs in HNSCC.

We selected 250 HNSCC samples as the training cohort and established an 8‐lncRNA prognostic model by using univariate, multivariate, and Lasso–Cox analyses. HNSCC patients were divided into high‐ and low‐risk groups according to the median risk score, and the high‐risk group was found to have worse prognosis than the low‐risk group.

In the training set, the prognostic diagnostic efficiency values of the 3‐ and 5‐year ROCs were 0.686 and 0.709, respectively. In the internal validation set, the prognostic diagnostic efficiency values of the 3‐ and 5‐year ROCs were 0.679 and 0.704, respectively. In the independent verification set, the prognostic diagnostic efficiency values of the 3‐ and 5‐year ROCs were 0.653 and 0.749, respectively. In the 5‐year prognostic classification, the average ROC of the model was > 0.7. Therefore, our lncRNA signature was more suitable for predicting the 5‐year survival rate of patients compared with the 3‐year survival rate.

The results in the testing cohort and external validation set were consistent with those in the training cohort, suggesting that our 8‐lncRNA signature had stable robustness and could well distinguish high‐risk patients from low‐risk patients.

We identified a co‐expressed lncRNA module closely related to survival status via WGCNA, and GO analysis results showed that the module was mainly involved in the biological processes of negative regulation of cell differentiation and the MAPK signaling pathway. Univariate and multivariate Cox regression analyses were conducted in the training and testing cohorts, and the results suggested that the 8‐lncRNA risk score could be used as an independent prognostic marker. The experimental results of PCR showed that compared to normal samples, PRKG1‐AS1 and SH3BP5‐AS1 were significantly upregulated while LINC00567, LINC00996, MTOR‐AS1, RAB11B‐AS1, RPS6KA2‐AS1, and ZNF451‐AS1 were significantly downregulated in tumor tissues.

Decreased LINC00996 expression is associated with the occurrence and metastasis of colorectal cancer, and LINC00996 depletion is associated with poor prognosis in patients with colorectal cancer, suggesting that LINC00996 may adjust the JAK‐STAT, NF‐κB, HIF‐1, TLR, and PI3K‐AKT signaling pathways to suppress tumor occurrence and metastasis [26]. High PRKG1‐AS1 expression in oral cancer is predictive of adverse outcomes [27]. RAB11B‐AS1 is significantly reduced in osteosarcoma, and it is associated with the metastasis and poor prognosis of osteosarcoma. Reduced RAB11B‐AS1 can significantly promote the proliferation, migration, and invasion of osteosarcoma cells; prevent the apoptosis of osteosarcoma cells; and lead to reduced cisplatin susceptibility. Moreover, upregulated RAB11B‐AS1 can inhibit human osteosarcoma cell attack [28]. SH3BP5‐AS1 is significantly upregulated in neuroblastoma [29]. MTOR‐AS1 is associated with cryptorchidism [30]. RPS6KA2‐AS1 is considered a potential biomarker of acute stroke and is involved in the neurotrophin signaling pathway [31]. To date, there have been no relevant studies on LINC00567, MTOR‐AS1, ZNF451‐AS1, or RPS6KA2‐AS1 in cancer.

To investigate the mechanisms of the 8 lncRNAs in the progression of HNSCC, GSEA was performed. The results showed that the low‐risk group was mainly enriched in natural killer cell‐mediated cytotoxicity and the phosphatidylinositol signaling system. Natural killer cells, which are a special type of white blood cell, can specifically recognize and destroy tumor cells [32]. Based on this mechanism, natural killer cell‐mediated tumor therapy has been developed clinically. Essentially, this means injecting natural killer cells into the body to destroy tumor tissues, as shown in a study in which tumor cells were removed from the bodies of patients with leukemia [33]. The phosphatidylinositol signaling system is a complex cellular regulatory system composed of enzymes, phospholipid messengers, and their binding proteins, and it plays an important regulatory role in cell growth, proliferation, survival, and cell movement [34]. Mutations in the enzyme that activates the phosphatidylinositol messenger lead to high activation of the phosphatidylinositol signaling system, resulting in abnormal cell proliferation, endocytosis, cell metastasis, and even tumorigenesis [35]. The phosphatidylinositol signaling system plays an important role in tumor proliferation and metastasis, so the components of the phosphatidylinositol system have the potential to become good clinical therapeutic targets. More and more drugs are on the pathway toward clinical use, for example, the phosphatidylinositol 3 kinase (PI3K) inhibitor wortmannin. Wortmannin and LY294002 can quickly target PI3K, inhibit tumor AKT phosphorylation, and prevent the activation of downstream growth signals [36, 37]. The mTOR inhibitor rapamycin targets mTOR and is highly effective in treating breast cancer, cervical cancer, and HNSCC [38, 39, 40]. The lower risk of HNSCC recurrence in the low‐risk group of this study may be related to the above mechanisms.

The study has several limitations. First, the existing clinical information was limited. Only tumor stage and grade data were available, and information about other important characteristics, such as tumor size, chemotherapy drug resistance, lymph node metastasis, and vascular invasion was missing, which may have affected the accuracy of the lncRNA risk score model. In addition, we predicted possible mechanisms, but lncRNA‐specific functions in HNSCC remain unclear, so more experiments are needed for verification.

In short, we constructed an 8‐lncRNA signature as a prognostic factor of HNSCC through Lasso and multivariable Cox analyses. GSEA results showed that the lncRNAs affected the progress of HNSCC through natural killer cell‐mediated cytotoxicity and the phosphatidylinositol signaling system.

The lncRNA‐based risk score prognostic model was used to evaluate patients' prognostic scores. When a patient’s risk score was > 0, the patient was considered high‐risk. Clinicians can use such information to change patients' treatment plans according to the predicted results of the model in order to realize the individualized treatment of patients with HNSCC. Strategies should be developed to prevent or detect HNSCC recurrence early in high‐risk groups. Therefore, high‐risk groups should be followed more frequently.

## Conflict of interest

The authors declare no conflict of interest.

## Author contributions

SL made substantial contributions to the conception, performed the experiments, and wrote and revised the manuscript.

## Supporting information

**Table S1.** The target genes regulated by the lncRNAs in the hub module.

## Data Availability

The data used to support the findings of this study are available from the corresponding author on reasonable request.
