# Supplementary material for: Identification of hub lncRNAs in head and neck cancer based on weighted gene co‐expression network analysis and experiments
Source: FEBS Open Bio. 2021 May 21;11(7):2060–73. doi: 10.1002/2211-5463.13134 (PMC8406479; doi:10.1002/2211-5463.13134)
Supplement: Supplementary file 1 — Table S1. The target genes regulated by the lncRNAs in the hub module. [file FEB4-11-2060-s001.docx]

**Target genes regulated by lncRNA**

lncRNA miRNA miRNA Gene miRDB miRTarBase TargetScan Sum

C9orf139 hsa-miR-7 hsa-miR-1297 GTF2A1 1 1 1 3

C9orf139 hsa-miR-7ab hsa-miR-125b-5p CDH5 1 1 1 3

C9orf139 hsa-miR-133abc hsa-miR-761 TRIM29 1 1 1 3

C9orf139 hsa-miR-138 hsa-miR-107 NOTCH2 1 1 1 3

C9orf139 hsa-miR-138ab hsa-miR-455-5p GSG1 1 1 1 3

C9orf139 hsa-miR-140 hsa-miR-17-5p PLXNA1 1 1 1 3

C9orf139 hsa-miR-140-5p hsa-miR-20b-5p RABEP1 1 1 1 3

C9orf139 hsa-miR-876-3p hsa-miR-301b-3p ZBTB4 1 1 1 3

C9orf139 hsa-miR-1244 hsa-miR-206 G6PD 1 1 1 3

C9orf139 hsa-miR-143 hsa-miR-17-5p ZNF532 1 1 1 3

C9orf139 hsa-miR-1721 hsa-miR-17-5p FJX1 1 1 1 3

C9orf139 hsa-miR-4770 hsa-miR-301b-3p DICER1 1 1 1 3

C9orf139 hsa-miR-150 hsa-miR-17-5p CTSA 1 1 1 3

C9orf139 hsa-miR-5127 hsa-miR-107 OTUD7B 1 1 1 3

C9orf139 hsa-miR-184 hsa-miR-20b-5p C14orf28 1 1 1 3

C9orf139 hsa-miR-187 hsa-miR-363-3p GEMIN2 1 1 1 3

C9orf139 hsa-miR-190 hsa-miR-107 TGFBR3 1 1 1 3

C9orf139 hsa-miR-190ab hsa-miR-22-3p TNFRSF10D 1 1 1 3

C9orf139 hsa-miR-1ab hsa-miR-490-3p MAP3K9 1 1 1 3

C9orf139 hsa-miR-206 hsa-miR-107 NUP50 1 1 1 3

C9orf139 hsa-miR-613 hsa-miR-20b-5p CEP97 1 1 1 3

C9orf139 hsa-miR-204 hsa-miR-17-5p PDZD11 1 1 1 3

C9orf139 hsa-miR-204b hsa-miR-20b-5p BRMS1L 1 1 1 3

C9orf139 hsa-miR-211 hsa-miR-301b-3p ARHGAP12 1 1 1 3

C9orf139 hsa-miR-205 hsa-miR-1297 MTMR12 1 1 1 3

C9orf139 hsa-miR-205ab hsa-miR-301b-3p MMGT1 1 1 1 3

C9orf139 hsa-miR-216b hsa-miR-125a-5p TMEM136 1 1 1 3

C9orf139 hsa-miR-216b-5p hsa-miR-3619-5p PIM1 1 1 1 3

C9orf139 hsa-miR-22 hsa-miR-17-5p ARHGAP12 1 1 1 3

C9orf139 hsa-miR-22-3p hsa-miR-20b-5p PARD6B 1 1 1 3

C9orf139 hsa-miR-122 hsa-miR-216b-5p KLF12 1 1 1 3

C9orf139 hsa-miR-122a hsa-miR-20b-5p FBXO48 1 1 1 3

C9orf139 hsa-miR-1352 hsa-miR-107 LATS2 1 1 1 3

C9orf139 hsa-miR-24 hsa-miR-24-3p PLIN3 1 1 1 3

C9orf139 hsa-miR-24ab hsa-miR-20b-5p PLXNA1 1 1 1 3

C9orf139 hsa-miR-24-3p hsa-miR-17-5p PRR14L 1 1 1 3

C9orf139 hsa-miR-26ab hsa-miR-24-3p INMT 1 1 1 3

C9orf139 hsa-miR-1297 hsa-miR-20b-5p BTBD7 1 1 1 3

C9orf139 hsa-miR-4465 hsa-miR-20b-5p NUP35 1 1 1 3

C9orf139 hsa-miR-101 hsa-miR-17-5p FAM129A 1 1 1 3

C9orf139 hsa-miR-101ab hsa-miR-20b-5p ANKH 1 1 1 3

C9orf139 hsa-miR-31 hsa-miR-20b-5p STX6 1 1 1 3

C9orf139 hsa-miR-103a hsa-miR-125b-5p SCARB2 1 1 1 3

C9orf139 hsa-miR-107 hsa-miR-22-3p TIAM1 1 1 1 3

C9orf139 hsa-miR-107ab hsa-miR-24-3p MIDN 1 1 1 3

C9orf139 hsa-miR-33ab hsa-miR-17-5p REST 1 1 1 3

C9orf139 hsa-miR-33-5p hsa-miR-363-3p EDEM1 1 1 1 3

C9orf139 hsa-miR-125a-5p hsa-miR-20b-5p RORA 1 1 1 3

C9orf139 hsa-miR-125b-5p hsa-miR-107 CNNM2 1 1 1 3

C9orf139 hsa-miR-351 hsa-miR-216b-5p COL4A4 1 1 1 3

C9orf139 hsa-miR-670 hsa-miR-23b-3p NUFIP2 1 1 1 3

C9orf139 hsa-miR-4319 hsa-miR-20b-5p ITCH 1 1 1 3

C9orf139 hsa-miR-10abc hsa-miR-17-5p ORMDL3 1 1 1 3

C9orf139 hsa-miR-10a-5p hsa-miR-301b-3p SERINC3 1 1 1 3

C9orf139 hsa-miR-128 hsa-miR-20b-5p KLF10 1 1 1 3

C9orf139 hsa-miR-128ab hsa-miR-363-3p BCL11B 1 1 1 3

C9orf139 hsa-miR-490-3p hsa-miR-24-3p ACVR1B 1 1 1 3

EGOT hsa-miR-135ab hsa-miR-455-5p MYLIP 1 1 1 3

EGOT hsa-miR-135a-5p hsa-miR-27a-3p SGMS1 1 1 1 3

EGOT hsa-miR-141 hsa-miR-23b-3p PTK2B 1 1 1 3

EGOT hsa-miR-200a hsa-miR-301b-3p HABP4 1 1 1 3

EGOT hsa-miR-143 hsa-miR-27a-3p EN2 1 1 1 3

EGOT hsa-miR-1721 hsa-miR-17-5p ABHD2 1 1 1 3

EGOT hsa-miR-4770 hsa-miR-20b-5p CHIC1 1 1 1 3

EGOT hsa-miR-146ac hsa-miR-1297 NUS1 1 1 1 3

EGOT hsa-miR-146b-5p hsa-miR-17-5p HSPA8 1 1 1 3

EGOT hsa-miR-15abc hsa-miR-3619-5p FAM49B 1 1 1 3

EGOT hsa-miR-16 hsa-miR-24-3p FBLIM1 1 1 1 3

EGOT hsa-miR-16abc hsa-miR-20b-5p JAK1 1 1 1 3

EGOT hsa-miR-195 hsa-miR-20b-5p RPS6KA5 1 1 1 3

EGOT hsa-miR-322 hsa-miR-107 VCAN 1 1 1 3

EGOT hsa-miR-424 hsa-miR-20b-5p SIK1 1 1 1 3

EGOT hsa-miR-497 hsa-miR-1297 OTUD4 1 1 1 3

EGOT hsa-miR-1907 hsa-miR-20b-5p MAPK1 1 1 1 3

EGOT hsa-miR-183 hsa-miR-17-5p RAB5B 1 1 1 3

EGOT hsa-miR-203 hsa-miR-24-3p SSR1 1 1 1 3

EGOT hsa-miR-205 hsa-miR-761 SCAMP4 1 1 1 3

EGOT hsa-miR-205ab hsa-miR-17-5p PTGFRN 1 1 1 3

EGOT hsa-miR-21 hsa-miR-10a-5p PAPD5 1 1 1 3

EGOT hsa-miR-590-5p hsa-miR-140-5p PRDM1 1 1 1 3

EGOT hsa-miR-214 hsa-miR-17-5p FOXJ3 1 1 1 3

EGOT hsa-miR-761 hsa-miR-24-3p PTPRF 1 1 1 3

EGOT hsa-miR-3619-5p hsa-miR-363-3p PTAR1 1 1 1 3

EGOT hsa-miR-23abc hsa-miR-10a-5p SON 1 1 1 3

EGOT hsa-miR-23b-3p hsa-miR-17-5p PPP3R1 1 1 1 3

EGOT hsa-miR-103a hsa-miR-363-3p TRIM36 1 1 1 3

EGOT hsa-miR-107 hsa-miR-206 ZNF215 1 1 1 3

EGOT hsa-miR-107ab hsa-miR-425-5p ATP5G3 1 1 1 3

EGOT hsa-miR-33ab hsa-miR-20b-5p TMEM245 1 1 1 3

EGOT hsa-miR-33-5p hsa-miR-17-5p MORF4L1 1 1 1 3

EGOT hsa-miR-375 hsa-miR-107 GCC1 1 1 1 3

EGOT hsa-miR-125a-5p hsa-miR-125a-5p MAP2K7 1 1 1 3

EGOT hsa-miR-125b-5p hsa-miR-20b-5p MCC 1 1 1 3

EGOT hsa-miR-351 hsa-miR-129-5p DUSP10 1 1 1 3

EGOT hsa-miR-670 hsa-miR-17-5p LIMK1 1 1 1 3

EGOT hsa-miR-4319 hsa-miR-10a-5p ELOVL2 1 1 1 3

EGOT hsa-miR-10abc hsa-miR-301b-3p ZNF711 1 1 1 3

EGOT hsa-miR-10a-5p hsa-miR-363-3p CPEB3 1 1 1 3

EGOT hsa-miR-129-5p hsa-miR-1297 PHAX 1 1 1 3

EGOT hsa-miR-129ab-5p hsa-miR-3619-5p SOCS5 1 1 1 3

EGOT hsa-miR-499-5p hsa-miR-20b-5p GBF1 1 1 1 3

LINC00426 hsa-miR-1378 hsa-miR-17-5p PKNOX1 1 1 1 3

LINC00426 hsa-miR-1420ac hsa-miR-1297 TET3 1 1 1 3

LINC00426 hsa-miR-96 hsa-miR-761 LUZP1 1 1 1 3

LINC00426 hsa-miR-507 hsa-miR-363-3p GTF2A1 1 1 1 3

LINC00426 hsa-miR-1271 hsa-miR-20b-5p CYBRD1 1 1 1 3

LINC00426 hsa-miR-138 hsa-miR-363-3p SESN3 1 1 1 3

LINC00426 hsa-miR-138ab hsa-miR-17-5p LDLR 1 1 1 3

LINC00426 hsa-miR-141 hsa-miR-761 NUFIP2 1 1 1 3

LINC00426 hsa-miR-200a hsa-miR-17-5p SIK1 1 1 1 3

LINC00426 hsa-miR-143 hsa-miR-27a-3p LITAF 1 1 1 3

LINC00426 hsa-miR-1721 hsa-miR-17-5p WAC 1 1 1 3

LINC00426 hsa-miR-4770 hsa-miR-125b-5p TMEM136 1 1 1 3

LINC00426 hsa-miR-145 hsa-miR-17-5p ITCH 1 1 1 3

LINC00426 hsa-miR-146ac hsa-miR-301b-3p DLG5 1 1 1 3

LINC00426 hsa-miR-146b-5p hsa-miR-1297 DEPDC1 1 1 1 3

LINC00426 hsa-miR-148ab-3p hsa-miR-107 CAB39 1 1 1 3

LINC00426 hsa-miR-152 hsa-miR-761 C17orf49 1 1 1 3

LINC00426 hsa-miR-150 hsa-miR-20b-5p WDR37 1 1 1 3

LINC00426 hsa-miR-5127 hsa-miR-20b-5p E2F1 1 1 1 3

LINC00426 hsa-miR-15abc hsa-miR-10a-5p XRN1 1 1 1 3

LINC00426 hsa-miR-16 hsa-miR-216b-5p TM9SF3 1 1 1 3

LINC00426 hsa-miR-16abc hsa-miR-10a-5p RPRD1A 1 1 1 3

LINC00426 hsa-miR-195 hsa-miR-17-5p PTPDC1 1 1 1 3

LINC00426 hsa-miR-322 hsa-miR-24-3p BCL7A 1 1 1 3

LINC00426 hsa-miR-424 hsa-miR-363-3p ARID1B 1 1 1 3

LINC00426 hsa-miR-497 hsa-miR-10a-5p HOXA3 1 1 1 3

LINC00426 hsa-miR-1907 hsa-miR-20b-5p RRM2 1 1 1 3

LINC00426 hsa-miR-17 hsa-miR-22-3p SERBP1 1 1 1 3

LINC00426 hsa-miR-17-5p hsa-miR-20b-5p TXLNA 1 1 1 3

LINC00426 hsa-miR-20ab hsa-miR-17-5p DNM1L 1 1 1 3

LINC00426 hsa-miR-20b-5p hsa-miR-24-3p PDXK 1 1 1 3

LINC00426 hsa-miR-106ab hsa-miR-20b-5p TMEM167A 1 1 1 3

LINC00426 hsa-miR-427 hsa-miR-17-5p DUSP2 1 1 1 3

LINC00426 hsa-miR-518a-3p hsa-miR-146b-5p ZNRF3 1 1 1 3

LINC00426 hsa-miR-519d hsa-miR-17-5p LPGAT1 1 1 1 3

LINC00426 hsa-miR-182 hsa-miR-17-5p YOD1 1 1 1 3

LINC00426 hsa-miR-183 hsa-miR-363-3p PDPN 1 1 1 3

LINC00426 hsa-miR-184 hsa-miR-20b-5p HIF1A 1 1 1 3

LINC00426 hsa-miR-190 hsa-miR-17-5p SCAMP5 1 1 1 3

LINC00426 hsa-miR-190ab hsa-miR-301b-3p DYNC1LI2 1 1 1 3

LINC00426 hsa-miR-192 hsa-miR-27a-3p ABHD17C 1 1 1 3

LINC00426 hsa-miR-215 hsa-miR-17-5p TOPORS 1 1 1 3

LINC00426 hsa-miR-193 hsa-miR-301b-3p BTF3L4 1 1 1 3

LINC00426 hsa-miR-193b hsa-miR-23b-3p PNRC2 1 1 1 3

LINC00426 hsa-miR-193a-3p hsa-miR-301b-3p LCLAT1 1 1 1 3

LINC00426 hsa-miR-194 hsa-miR-17-5p FNBP1L 1 1 1 3

LINC00426 hsa-miR-199ab-5p hsa-miR-17-5p NPAS2 1 1 1 3

LINC00426 hsa-miR-4319 hsa-miR-17-5p FAM46C 1 1 1 3

LINC00426 hsa-miR-455-5p hsa-miR-20b-5p PFKP 1 1 1 3

LINC00426 hsa-miR-128 hsa-miR-125b-5p IKZF4 1 1 1 3

LINC00426 hsa-miR-128ab hsa-miR-17-5p TGFBR2 1 1 1 3

LINC00426 hsa-miR-129-5p hsa-miR-490-3p PAPPA 1 1 1 3

LINC00426 hsa-miR-129ab-5p hsa-miR-129-5p BMPR2 1 1 1 3

LINC00426 hsa-miR-499-5p hsa-miR-17-5p CASP2 1 1 1 3

LINC01729 hsa-miR-130ac hsa-miR-425-5p OCRL 1 1 1 3

LINC01729 hsa-miR-301ab hsa-miR-27a-3p NLN 1 1 1 3

LINC01729 hsa-miR-301b hsa-miR-17-5p CFL2 1 1 1 3

LINC01729 hsa-miR-301b-3p hsa-miR-24-3p DYRK2 1 1 1 3

LINC01729 hsa-miR-454 hsa-miR-125b-5p SAMD10 1 1 1 3

LINC01729 hsa-miR-721 hsa-miR-17-5p GNS 1 1 1 3

LINC01729 hsa-miR-4295 hsa-miR-129-5p DIS3 1 1 1 3

LINC01729 hsa-miR-3666 hsa-miR-363-3p CNEP1R1 1 1 1 3

LINC01729 hsa-miR-9 hsa-miR-125a-5p LFNG 1 1 1 3

LINC01729 hsa-miR-9ab hsa-miR-20b-5p FOXQ1 1 1 1 3

LINC01729 hsa-miR-93 hsa-miR-216b-5p CCDC65 1 1 1 3

LINC01729 hsa-miR-93a hsa-miR-27a-3p ZNF800 1 1 1 3

LINC01729 hsa-miR-105 hsa-miR-425-5p BHLHB9 1 1 1 3

LINC01729 hsa-miR-106a hsa-miR-27a-3p ZNF148 1 1 1 3

LINC01729 hsa-miR-291a-3p hsa-miR-613 CERS2 1 1 1 3

LINC01729 hsa-miR-294 hsa-miR-107 BAZ2A 1 1 1 3

LINC01729 hsa-miR-295 hsa-miR-17-5p CRY2 1 1 1 3

LINC01729 hsa-miR-302abcde hsa-miR-24-3p SCML1 1 1 1 3

LINC01729 hsa-miR-372 hsa-miR-20b-5p SLC16A9 1 1 1 3

LINC01729 hsa-miR-373 hsa-miR-129-5p RBM47 1 1 1 3

LINC01729 hsa-miR-428 hsa-miR-363-3p VPS4B 1 1 1 3

LINC01729 hsa-miR-519a hsa-miR-20b-5p TGFBR2 1 1 1 3

LINC01729 hsa-miR-520be hsa-miR-20b-5p ZNF280B 1 1 1 3

LINC01729 hsa-miR-520acd-3p hsa-miR-301b-3p BLCAP 1 1 1 3

LINC01772 hsa-miR-1ab hsa-miR-1297 CKS2 1 1 1 3

LINC01772 hsa-miR-206 hsa-miR-3619-5p AMER1 1 1 1 3

LINC01772 hsa-miR-613 hsa-miR-20b-5p TNKS2 1 1 1 3

LINC01772 hsa-miR-203 hsa-miR-17-5p DDHD1 1 1 1 3

LINC01772 hsa-miR-205 hsa-miR-24-3p NDST1 1 1 1 3

LINC01772 hsa-miR-205ab hsa-miR-425-5p SYNCRIP 1 1 1 3

LINC01772 hsa-miR-208ab hsa-miR-125a-5p SCARB2 1 1 1 3

LINC01772 hsa-miR-208ab-3p hsa-miR-20b-5p EIF4H 1 1 1 3

LINC01772 hsa-miR-210 hsa-miR-20b-5p TANC1 1 1 1 3

LINC01772 hsa-miR-214 hsa-miR-17-5p KIF23 1 1 1 3

LINC01772 hsa-miR-761 hsa-miR-20b-5p EEA1 1 1 1 3

LINC01772 hsa-miR-3619-5p hsa-miR-761 CDC42SE1 1 1 1 3

LINC01772 hsa-miR-216b hsa-miR-24-3p C15orf39 1 1 1 3

LINC01772 hsa-miR-216b-5p hsa-miR-301b-3p SLC12A7 1 1 1 3

LINC01772 hsa-miR-221 hsa-miR-17-5p TMEM138 1 1 1 3

LINC01772 hsa-miR-222 hsa-miR-17-5p USP3 1 1 1 3

LINC01772 hsa-miR-222ab hsa-miR-17-5p CADM2 1 1 1 3

LINC01772 hsa-miR-1928 hsa-miR-23b-3p GJA1 1 1 1 3

LINC01772 hsa-miR-122 hsa-miR-20b-5p CCDC71L 1 1 1 3

LINC01772 hsa-miR-122a hsa-miR-23b-3p MARCKS 1 1 1 3

LINC01772 hsa-miR-1352 hsa-miR-27a-3p MED14 1 1 1 3

LINC01772 hsa-miR-23abc hsa-miR-20b-5p PPP1R15B 1 1 1 3

LINC01772 hsa-miR-23b-3p hsa-miR-761 SOCS5 1 1 1 3

LINC01772 hsa-miR-24 hsa-miR-20b-5p STK17B 1 1 1 3

LINC01772 hsa-miR-24ab hsa-miR-24-3p MAGI1 1 1 1 3

LINC01772 hsa-miR-24-3p hsa-miR-3619-5p NUFIP2 1 1 1 3

LINC01772 hsa-miR-25 hsa-miR-22-3p DDIT4 1 1 1 3

LINC01772 hsa-miR-32 hsa-miR-129-5p USP6NL 1 1 1 3

LINC01772 hsa-miR-92abc hsa-miR-3619-5p MPDU1 1 1 1 3

LINC01772 hsa-miR-363 hsa-miR-24-3p UBE2K 1 1 1 3

LINC01772 hsa-miR-363-3p hsa-miR-490-3p RBPJ 1 1 1 3

LINC01772 hsa-miR-367 hsa-miR-1297 C14orf37 1 1 1 3

LINC01772 hsa-miR-27abc hsa-miR-17-5p HIP1 1 1 1 3

LINC01772 hsa-miR-27a-3p hsa-miR-125b-5p LIPA 1 1 1 3

LINC01772 hsa-miR-29abcd hsa-miR-20b-5p DDHD1 1 1 1 3

LINC01772 hsa-miR-103a hsa-miR-24-3p TOP1 1 1 1 3

LINC01772 hsa-miR-107 hsa-miR-17-5p ANKIB1 1 1 1 3

LINC01772 hsa-miR-107ab hsa-miR-125b-5p EIF1AD 1 1 1 3

LINC01772 hsa-miR-124 hsa-miR-27a-3p ITSN2 1 1 1 3

LINC01772 hsa-miR-124ab hsa-miR-363-3p GFPT2 1 1 1 3

LINC01772 hsa-miR-506 hsa-miR-17-5p ANKRD33B 1 1 1 3

LINC01772 hsa-miR-338 hsa-miR-17-5p FAM102A 1 1 1 3

LINC01772 hsa-miR-338-3p hsa-miR-17-5p U2SURP 1 1 1 3

LINC01772 hsa-miR-33ab hsa-miR-20b-5p PTPDC1 1 1 1 3

LINC01772 hsa-miR-33-5p hsa-miR-107 CSNK1G2 1 1 1 3

LINC01772 hsa-miR-425 hsa-miR-23b-3p TNRC6A 1 1 1 3

LINC01772 hsa-miR-425-5p hsa-miR-27a-3p SRSF1 1 1 1 3

LINC01772 hsa-miR-489 hsa-miR-425-5p EOGT 1 1 1 3

LINC01772 hsa-miR-125a-5p hsa-miR-301b-3p BRWD1 1 1 1 3

LINC01772 hsa-miR-125b-5p hsa-miR-17-5p USP32 1 1 1 3

LINC01772 hsa-miR-351 hsa-miR-301b-3p ZBTB18 1 1 1 3

LINC01772 hsa-miR-670 hsa-miR-363-3p EXOC5 1 1 1 3

MORC2-AS1 hsa-miR-7 hsa-miR-129-5p LIMS1 1 1 1 3

MORC2-AS1 hsa-miR-7ab hsa-miR-27a-3p GATA2 1 1 1 3

MORC2-AS1 hsa-miR-133abc hsa-miR-301b-3p RRAGD 1 1 1 3

MORC2-AS1 hsa-miR-138 hsa-miR-17-5p TMEM167A 1 1 1 3

MORC2-AS1 hsa-miR-138ab hsa-miR-27a-3p PLXND1 1 1 1 3

MORC2-AS1 hsa-miR-155 hsa-miR-20b-5p FRS2 1 1 1 3

MORC2-AS1 hsa-miR-18ab hsa-miR-27a-3p CAB39 1 1 1 3

MORC2-AS1 hsa-miR-4735-3p hsa-miR-363-3p SERTAD3 1 1 1 3

MORC2-AS1 hsa-miR-193 hsa-miR-20b-5p SOX4 1 1 1 3

MORC2-AS1 hsa-miR-193b hsa-miR-17-5p UBXN2A 1 1 1 3

MORC2-AS1 hsa-miR-193a-3p hsa-miR-17-5p ENPP5 1 1 1 3

MORC2-AS1 hsa-miR-199ab-5p hsa-miR-301b-3p CEP170 1 1 1 3

MORC2-AS1 hsa-miR-1ab hsa-miR-20b-5p FAM46C 1 1 1 3

MORC2-AS1 hsa-miR-206 hsa-miR-3619-5p GABARAP 1 1 1 3

MORC2-AS1 hsa-miR-613 hsa-miR-490-3p SMARCD1 1 1 1 3

MORC2-AS1 hsa-miR-214 hsa-miR-129-5p RBMXL1 1 1 1 3

MORC2-AS1 hsa-miR-761 hsa-miR-17-5p IFNAR1 1 1 1 3

MORC2-AS1 hsa-miR-3619-5p hsa-miR-27a-3p TGFBR1 1 1 1 3

MORC2-AS1 hsa-miR-216b hsa-miR-17-5p MTF1 1 1 1 3

MORC2-AS1 hsa-miR-216b-5p hsa-miR-425-5p DICER1 1 1 1 3

MORC2-AS1 hsa-miR-219-5p hsa-miR-301b-3p SMOC1 1 1 1 3

MORC2-AS1 hsa-miR-508 hsa-miR-301b-3p PRKAA1 1 1 1 3

MORC2-AS1 hsa-miR-508-3p hsa-miR-1297 RHOQ 1 1 1 3

MORC2-AS1 hsa-miR-4782-3p hsa-miR-10a-5p LIX1L 1 1 1 3

MORC2-AS1 hsa-miR-22 hsa-miR-20b-5p TMEM64 1 1 1 3

MORC2-AS1 hsa-miR-22-3p hsa-miR-22-3p TPD52L2 1 1 1 3

MORC2-AS1 hsa-miR-26ab hsa-miR-206 FNDC3A 1 1 1 3

MORC2-AS1 hsa-miR-1297 hsa-miR-363-3p ZDHHC5 1 1 1 3

MORC2-AS1 hsa-miR-4465 hsa-miR-20b-5p ZFYVE9 1 1 1 3

MORC2-AS1 hsa-miR-103a hsa-miR-17-5p FBXO28 1 1 1 3

MORC2-AS1 hsa-miR-107 hsa-miR-507 MAPK8 1 1 1 3

MORC2-AS1 hsa-miR-107ab hsa-miR-20b-5p PAFAH1B1 1 1 1 3

MORC2-AS1 hsa-miR-124 hsa-miR-613 HSP90B1 1 1 1 3

MORC2-AS1 hsa-miR-124ab hsa-miR-125b-5p ULK3 1 1 1 3

MORC2-AS1 hsa-miR-506 hsa-miR-107 SMARCE1 1 1 1 3

MORC2-AS1 hsa-miR-338 hsa-miR-22-3p C5orf24 1 1 1 3

MORC2-AS1 hsa-miR-338-3p hsa-miR-27a-3p RGPD4 1 1 1 3

MORC2-AS1 hsa-miR-455-5p hsa-miR-125b-5p SEMA4C 1 1 1 3

MORC2-AS1 hsa-miR-490-3p hsa-miR-17-5p MSMO1 1 1 1 3

MTOR-AS1 hsa-miR-137 hsa-miR-107 OGT 1 1 1 3

MTOR-AS1 hsa-miR-137ab hsa-miR-17-5p BICD2 1 1 1 3

MTOR-AS1 hsa-miR-145 hsa-miR-27a-3p NR1D2 1 1 1 3

MTOR-AS1 hsa-miR-1ab hsa-miR-301b-3p SIK1 1 1 1 3

MTOR-AS1 hsa-miR-206 hsa-miR-20b-5p PIP4K2A 1 1 1 3

MTOR-AS1 hsa-miR-613 hsa-miR-17-5p GIGYF1 1 1 1 3

MTOR-AS1 hsa-miR-205 hsa-miR-129-5p CNTLN 1 1 1 3

MTOR-AS1 hsa-miR-205ab hsa-miR-17-5p KLF3 1 1 1 3

MTOR-AS1 hsa-miR-223 hsa-miR-20b-5p LAPTM4A 1 1 1 3

MTOR-AS1 hsa-miR-122 hsa-miR-22-3p ERBB3 1 1 1 3

MTOR-AS1 hsa-miR-122a hsa-miR-206 PAX3 1 1 1 3

MTOR-AS1 hsa-miR-1352 hsa-miR-107 CDK8 1 1 1 3

MTOR-AS1 hsa-miR-33a-3p hsa-miR-23b-3p RPRD2 1 1 1 3

MTOR-AS1 hsa-miR-365 hsa-miR-27a-3p TMBIM6 1 1 1 3

MTOR-AS1 hsa-miR-365-3p hsa-miR-140-5p PHACTR2 1 1 1 3

MTOR-AS1 hsa-miR-375 hsa-miR-17-5p CYBRD1 1 1 1 3

MTOR-AS1 hsa-miR-10abc hsa-miR-17-5p ELK4 1 1 1 3

MTOR-AS1 hsa-miR-10a-5p hsa-miR-3619-5p C17orf49 1 1 1 3

MTOR-AS1 hsa-miR-490-3p hsa-miR-193a-3p DCAF7 1 1 1 3

SEC62-AS1 hsa-miR-9 hsa-miR-184 LRRC8A 1 1 1 3

SEC62-AS1 hsa-miR-9ab hsa-miR-301b-3p PAPD4 1 1 1 3

SEC62-AS1 hsa-miR-93 hsa-miR-301b-3p ZMAT3 1 1 1 3

SEC62-AS1 hsa-miR-93a hsa-miR-17-5p HMBOX1 1 1 1 3

SEC62-AS1 hsa-miR-105 hsa-miR-17-5p PFKP 1 1 1 3

SEC62-AS1 hsa-miR-106a hsa-miR-301b-3p SECISBP2L 1 1 1 3

SEC62-AS1 hsa-miR-291a-3p hsa-miR-216b-5p SOCS6 1 1 1 3

SEC62-AS1 hsa-miR-294 hsa-miR-17-5p BTN3A1 1 1 1 3

SEC62-AS1 hsa-miR-295 hsa-miR-301b-3p ARHGAP1 1 1 1 3

SEC62-AS1 hsa-miR-302abcde hsa-miR-20b-5p CRIM1 1 1 1 3

SEC62-AS1 hsa-miR-372 hsa-miR-20b-5p KIAA0513 1 1 1 3

SEC62-AS1 hsa-miR-373 hsa-miR-125a-5p EIF1AD 1 1 1 3

SEC62-AS1 hsa-miR-428 hsa-miR-17-5p ELAVL2 1 1 1 3

SEC62-AS1 hsa-miR-519a hsa-miR-301b-3p CFL2 1 1 1 3

SEC62-AS1 hsa-miR-520be hsa-miR-17-5p VPS13C 1 1 1 3

SEC62-AS1 hsa-miR-520acd-3p hsa-miR-20b-5p RGMB 1 1 1 3

SEC62-AS1 hsa-miR-1378 hsa-miR-20b-5p RUNX3 1 1 1 3

SEC62-AS1 hsa-miR-1420ac hsa-miR-20b-5p GOLGA1 1 1 1 3

SEC62-AS1 hsa-miR-17 hsa-miR-20b-5p TSG101 1 1 1 3

SEC62-AS1 hsa-miR-17-5p hsa-miR-20b-5p ZNF202 1 1 1 3

SEC62-AS1 hsa-miR-20ab hsa-miR-24-3p DND1 1 1 1 3

SEC62-AS1 hsa-miR-20b-5p hsa-miR-17-5p ABCA1 1 1 1 3

SEC62-AS1 hsa-miR-106ab hsa-miR-20b-5p RAB11FIP1 1 1 1 3

SEC62-AS1 hsa-miR-427 hsa-miR-24-3p PTPN9 1 1 1 3

SEC62-AS1 hsa-miR-518a-3p hsa-miR-20b-5p CMPK1 1 1 1 3

SEC62-AS1 hsa-miR-519d hsa-miR-17-5p MTMR3 1 1 1 3

hsa-miR-363-3p TOB1 1 1 1 3

hsa-miR-17-5p F3 1 1 1 3

hsa-miR-17-5p ZNF800 1 1 1 3

hsa-miR-17-5p NAGK 1 1 1 3

hsa-miR-363-3p FOXN2 1 1 1 3

hsa-miR-1244 DCK 1 1 1 3

hsa-miR-17-5p CRK 1 1 1 3

hsa-miR-3619-5p PNPLA6 1 1 1 3

hsa-miR-17-5p LIMA1 1 1 1 3

hsa-miR-125b-5p ETS1 1 1 1 3

hsa-miR-20b-5p EIF5A2 1 1 1 3

hsa-miR-17-5p RB1 1 1 1 3

hsa-miR-761 ZBTB10 1 1 1 3

hsa-miR-20b-5p CDKN1A 1 1 1 3

hsa-miR-20b-5p BTG3 1 1 1 3

hsa-miR-301b-3p DPYSL2 1 1 1 3

hsa-miR-20b-5p CNOT7 1 1 1 3

hsa-miR-20b-5p PXK 1 1 1 3

hsa-miR-17-5p PAFAH1B1 1 1 1 3

hsa-miR-17-5p SSX2IP 1 1 1 3

hsa-miR-1297 E2F7 1 1 1 3

hsa-miR-425-5p CREBZF 1 1 1 3

hsa-miR-363-3p HIVEP1 1 1 1 3

hsa-miR-129-5p EXPH5 1 1 1 3

hsa-miR-129-5p STON2 1 1 1 3

hsa-miR-20b-5p FAM102A 1 1 1 3

hsa-miR-146b-5p MMP16 1 1 1 3

hsa-miR-125a-5p FBXO10 1 1 1 3

hsa-miR-1297 TOB1 1 1 1 3

hsa-miR-17-5p SRSF2 1 1 1 3

hsa-miR-27a-3p SUCO 1 1 1 3

hsa-miR-301b-3p NRBF2 1 1 1 3

hsa-miR-24-3p PIM2 1 1 1 3

hsa-miR-125b-5p TRIM71 1 1 1 3

hsa-miR-301b-3p ACVR1 1 1 1 3

hsa-miR-17-5p POLR3G 1 1 1 3

hsa-miR-17-5p MAPK1 1 1 1 3

hsa-miR-1297 ZNF608 1 1 1 3

hsa-miR-125a-5p SEMA4C 1 1 1 3

hsa-miR-17-5p PLEKHM1 1 1 1 3

hsa-miR-761 TRAF1 1 1 1 3

hsa-miR-24-3p EMP2 1 1 1 3

hsa-miR-301b-3p STX6 1 1 1 3

hsa-miR-23b-3p TNPO1 1 1 1 3

hsa-miR-107 PPP6C 1 1 1 3

hsa-miR-24-3p DDN 1 1 1 3

hsa-miR-27a-3p TNPO1 1 1 1 3

hsa-miR-107 CDK6 1 1 1 3

hsa-miR-24-3p ADPGK 1 1 1 3

hsa-miR-27a-3p PEG10 1 1 1 3

hsa-miR-125b-5p MKNK2 1 1 1 3

hsa-miR-761 JAG2 1 1 1 3

hsa-miR-17-5p FAXC 1 1 1 3

hsa-miR-23b-3p ZEB1 1 1 1 3

hsa-miR-20b-5p NKIRAS1 1 1 1 3

hsa-miR-107 PNISR 1 1 1 3

hsa-miR-27a-3p PDS5B 1 1 1 3

hsa-miR-17-5p OCRL 1 1 1 3

hsa-miR-20b-5p EPHA4 1 1 1 3

hsa-miR-20b-5p POLR3G 1 1 1 3

hsa-miR-27a-3p TMED5 1 1 1 3

hsa-miR-363-3p BCL2L11 1 1 1 3

hsa-miR-23b-3p FBN2 1 1 1 3

hsa-miR-613 WEE1 1 1 1 3

hsa-miR-20b-5p NETO2 1 1 1 3

hsa-miR-20b-5p PGM2L1 1 1 1 3

hsa-miR-125b-5p BMF 1 1 1 3

hsa-miR-27a-3p DNAJC27 1 1 1 3

hsa-miR-17-5p RNF145 1 1 1 3

hsa-miR-20b-5p GID4 1 1 1 3

hsa-miR-20b-5p HBP1 1 1 1 3

hsa-miR-20b-5p MORF4L1 1 1 1 3

hsa-miR-301b-3p RPS6KA5 1 1 1 3

hsa-miR-107 ABL2 1 1 1 3

hsa-miR-107 CLIP1 1 1 1 3

hsa-miR-27a-3p ZFHX3 1 1 1 3

hsa-miR-301b-3p ZFYVE9 1 1 1 3

hsa-miR-363-3p ZFC3H1 1 1 1 3

hsa-miR-193a-3p YWHAZ 1 1 1 3

hsa-miR-20b-5p PLEKHO2 1 1 1 3

hsa-miR-17-5p KPNA2 1 1 1 3

hsa-miR-17-5p ANKH 1 1 1 3

hsa-miR-17-5p GID4 1 1 1 3

hsa-miR-17-5p TNFRSF21 1 1 1 3

hsa-miR-363-3p GALNT7 1 1 1 3

hsa-miR-1297 UBR3 1 1 1 3

hsa-miR-20b-5p WAC 1 1 1 3

hsa-miR-23b-3p ETNK1 1 1 1 3

hsa-miR-301b-3p PHF12 1 1 1 3

hsa-miR-20b-5p CCL1 1 1 1 3

hsa-miR-363-3p GOLGA8J 1 1 1 3

hsa-miR-206 SMARCB1 1 1 1 3

hsa-miR-363-3p MFF 1 1 1 3

hsa-miR-24-3p MT1E 1 1 1 3

hsa-miR-507 CBX4 1 1 1 3

hsa-miR-22-3p ESR1 1 1 1 3

hsa-miR-17-5p MFSD8 1 1 1 3

hsa-miR-17-5p PHTF2 1 1 1 3

hsa-miR-17-5p CAPN15 1 1 1 3

hsa-miR-17-5p DDX5 1 1 1 3

hsa-miR-17-5p SEMA4B 1 1 1 3

hsa-miR-206 VAMP2 1 1 1 3

hsa-miR-301b-3p PPP1R15B 1 1 1 3

hsa-miR-23b-3p MMGT1 1 1 1 3

hsa-miR-301b-3p TRPC3 1 1 1 3

hsa-miR-24-3p PAK4 1 1 1 3

hsa-miR-24-3p VCPIP1 1 1 1 3

hsa-miR-20b-5p SUCO 1 1 1 3

hsa-miR-301b-3p MAPRE3 1 1 1 3

hsa-miR-17-5p MLXIP 1 1 1 3

hsa-miR-17-5p DENND5B 1 1 1 3

hsa-miR-301b-3p WASL 1 1 1 3

hsa-miR-17-5p PLEKHO2 1 1 1 3

hsa-miR-338-3p ACVR1 1 1 1 3

hsa-miR-363-3p SPRYD4 1 1 1 3

hsa-miR-23b-3p CA2 1 1 1 3

hsa-miR-17-5p AGO1 1 1 1 3

hsa-miR-206 MATR3 1 1 1 3

hsa-miR-125b-5p TOR2A 1 1 1 3

hsa-miR-301b-3p RAB14 1 1 1 3

hsa-miR-20b-5p RRAGD 1 1 1 3

hsa-miR-17-5p ANKFY1 1 1 1 3

hsa-miR-20b-5p DNAJC27 1 1 1 3

hsa-miR-129-5p PAPD5 1 1 1 3

hsa-miR-17-5p EIF4G2 1 1 1 3

hsa-miR-507 CLOCK 1 1 1 3

hsa-miR-761 MPDU1 1 1 1 3

hsa-miR-146b-5p IRAK1 1 1 1 3

hsa-miR-23b-3p PDIA6 1 1 1 3

hsa-miR-17-5p RBL1 1 1 1 3

hsa-miR-140-5p VEZF1 1 1 1 3

hsa-miR-20b-5p CENPQ 1 1 1 3

hsa-miR-125b-5p TBC1D1 1 1 1 3

hsa-miR-20b-5p FCHO2 1 1 1 3

hsa-miR-20b-5p FAM117B 1 1 1 3

hsa-miR-20b-5p NPAT 1 1 1 3

hsa-miR-206 LRRC59 1 1 1 3

hsa-miR-24-3p HKR1 1 1 1 3

hsa-miR-17-5p E2F2 1 1 1 3

hsa-miR-613 EIF1AX 1 1 1 3

hsa-miR-20b-5p NACC2 1 1 1 3

hsa-miR-27a-3p USP46 1 1 1 3

hsa-miR-17-5p BNIP2 1 1 1 3

hsa-miR-17-5p JAK1 1 1 1 3

hsa-miR-27a-3p ALG9 1 1 1 3

hsa-miR-3619-5p CS 1 1 1 3

hsa-miR-363-3p TULP4 1 1 1 3

hsa-miR-17-5p RBL2 1 1 1 3

hsa-miR-17-5p SPOPL 1 1 1 3

hsa-miR-301b-3p LMLN 1 1 1 3

hsa-miR-3619-5p SCAMP4 1 1 1 3

hsa-miR-17-5p FOXJ2 1 1 1 3

hsa-miR-20b-5p USP3 1 1 1 3

hsa-miR-17-5p E2F1 1 1 1 3

hsa-miR-27a-3p PDIA5 1 1 1 3

hsa-miR-363-3p DUSP5 1 1 1 3

hsa-miR-17-5p RPS6KA5 1 1 1 3

hsa-miR-20b-5p TRIP10 1 1 1 3

hsa-miR-206 SFRP1 1 1 1 3

hsa-miR-363-3p GOLGA8B 1 1 1 3

hsa-miR-24-3p TMEM209 1 1 1 3

hsa-miR-1297 TMEM2 1 1 1 3

hsa-miR-17-5p CDKN1A 1 1 1 3

hsa-miR-17-5p C7orf43 1 1 1 3

hsa-miR-507 BRD4 1 1 1 3

hsa-miR-17-5p HBP1 1 1 1 3

hsa-miR-507 CCNT2 1 1 1 3

hsa-miR-17-5p GINS4 1 1 1 3

hsa-miR-20b-5p ZBTB7A 1 1 1 3

hsa-miR-107 PIK3R1 1 1 1 3

hsa-miR-206 UTRN 1 1 1 3

hsa-miR-425-5p THRB 1 1 1 3

hsa-miR-107 FGF2 1 1 1 3

hsa-miR-17-5p NIN 1 1 1 3

hsa-miR-10a-5p MED1 1 1 1 3

hsa-miR-20b-5p CCND1 1 1 1 3

hsa-miR-206 ANP32B 1 1 1 3

hsa-miR-129-5p CDK6 1 1 1 3

hsa-miR-24-3p MAPK14 1 1 1 3

hsa-miR-17-5p UXS1 1 1 1 3

hsa-miR-20b-5p TP53INP1 1 1 1 3

hsa-miR-17-5p EPS15L1 1 1 1 3

hsa-miR-125b-5p CYP24A1 1 1 1 3

hsa-miR-27a-3p EIF5A2 1 1 1 3

hsa-miR-27a-3p HOXA10 1 1 1 3

hsa-miR-3619-5p KCTD15 1 1 1 3

hsa-miR-23b-3p POM121C 1 1 1 3

hsa-miR-20b-5p F3 1 1 1 3

hsa-miR-125b-5p EIF5A2 1 1 1 3

hsa-miR-140-5p CAPN1 1 1 1 3

hsa-miR-27a-3p AFF4 1 1 1 3

hsa-miR-216b-5p AKIP1 1 1 1 3

hsa-miR-17-5p UNK 1 1 1 3

hsa-miR-140-5p RPUSD2 1 1 1 3

hsa-miR-17-5p FAM117B 1 1 1 3

hsa-miR-27a-3p MESDC1 1 1 1 3

hsa-miR-17-5p NPAS3 1 1 1 3

hsa-miR-20b-5p ANKRD33B 1 1 1 3

hsa-miR-125a-5p TBC1D1 1 1 1 3

hsa-miR-1297 UGGT1 1 1 1 3

hsa-miR-3619-5p LUZP1 1 1 1 3

hsa-miR-24-3p NCOA5 1 1 1 3

hsa-miR-24-3p SCML2 1 1 1 3

hsa-miR-17-5p RAP2C 1 1 1 3

hsa-miR-27a-3p MIER3 1 1 1 3

hsa-miR-1297 BLOC1S2 1 1 1 3

hsa-miR-17-5p CHD9 1 1 1 3

hsa-miR-20b-5p NCOA3 1 1 1 3

hsa-miR-20b-5p DUSP2 1 1 1 3

hsa-miR-129-5p NWD1 1 1 1 3

hsa-miR-363-3p PHLPP2 1 1 1 3

hsa-miR-125a-5p TOR2A 1 1 1 3

hsa-miR-125b-5p ZFYVE1 1 1 1 3

hsa-miR-17-5p SLAIN2 1 1 1 3

hsa-miR-301b-3p PTPRG 1 1 1 3

hsa-miR-20b-5p SMAD5 1 1 1 3

hsa-miR-761 FLOT2 1 1 1 3

hsa-miR-20b-5p ANKIB1 1 1 1 3

hsa-miR-129-5p RYBP 1 1 1 3

hsa-miR-17-5p VPS26A 1 1 1 3

hsa-miR-17-5p EPHA4 1 1 1 3

hsa-miR-20b-5p CAPRIN2 1 1 1 3

hsa-miR-1297 EPB41L3 1 1 1 3

hsa-miR-20b-5p TOPORS 1 1 1 3

hsa-miR-27a-3p NR2F2 1 1 1 3

hsa-miR-17-5p TXLNA 1 1 1 3

hsa-miR-20b-5p ANKRD13C 1 1 1 3

hsa-miR-193a-3p GDF11 1 1 1 3

hsa-miR-17-5p TBC1D15 1 1 1 3

hsa-miR-24-3p TSC22D2 1 1 1 3

hsa-miR-363-3p TBC1D8 1 1 1 3

hsa-miR-107 TARBP2 1 1 1 3

hsa-miR-20b-5p TMBIM6 1 1 1 3

hsa-miR-140-5p TGFBR1 1 1 1 3

hsa-miR-17-5p WEE1 1 1 1 3

hsa-miR-20b-5p LASP1 1 1 1 3

hsa-miR-22-3p EDC3 1 1 1 3

hsa-miR-20b-5p UXS1 1 1 1 3

hsa-miR-301b-3p ARHGEF26 1 1 1 3

hsa-miR-17-5p TANC1 1 1 1 3

hsa-miR-20b-5p SPRED1 1 1 1 3

hsa-miR-1297 CHAC1 1 1 1 3

hsa-miR-125b-5p MFHAS1 1 1 1 3

hsa-miR-107 YTHDC1 1 1 1 3

hsa-miR-761 TFAP2C 1 1 1 3

hsa-miR-27a-3p CSRP2 1 1 1 3

hsa-miR-107 CALU 1 1 1 3

hsa-miR-125b-5p IL6R 1 1 1 3

hsa-miR-22-3p ARPC5 1 1 1 3

hsa-miR-338-3p NRP1 1 1 1 3

hsa-miR-20b-5p ULK1 1 1 1 3

hsa-miR-129-5p EBF1 1 1 1 3

hsa-miR-455-5p IPO7 1 1 1 3

hsa-miR-17-5p SLK 1 1 1 3

hsa-miR-17-5p NHLRC3 1 1 1 3

hsa-miR-425-5p FOXJ3 1 1 1 3

hsa-miR-17-5p FAM126B 1 1 1 3

hsa-miR-27a-3p RAP1B 1 1 1 3

hsa-miR-17-5p SSH2 1 1 1 3

hsa-miR-20b-5p RBBP7 1 1 1 3

hsa-miR-129-5p HNRNPA3 1 1 1 3

hsa-miR-27a-3p ADD1 1 1 1 3

hsa-miR-20b-5p NR2C2 1 1 1 3

hsa-miR-23b-3p LBR 1 1 1 3

hsa-miR-17-5p KIAA0513 1 1 1 3

hsa-miR-17-5p LCOR 1 1 1 3

hsa-miR-107 ZNF449 1 1 1 3

hsa-miR-20b-5p RBM20 1 1 1 3

hsa-miR-20b-5p CHAF1A 1 1 1 3

hsa-miR-17-5p GAB1 1 1 1 3

hsa-miR-20b-5p E2F2 1 1 1 3

hsa-miR-129-5p C1S 1 1 1 3

hsa-miR-140-5p PDGFRA 1 1 1 3

hsa-miR-20b-5p USP32 1 1 1 3

hsa-miR-20b-5p USP28 1 1 1 3

hsa-miR-301b-3p FBXO28 1 1 1 3

hsa-miR-20b-5p RB1 1 1 1 3

hsa-miR-876-3p YWHAE 1 1 1 3

hsa-miR-761 ZNF641 1 1 1 3

hsa-miR-107 DICER1 1 1 1 3

hsa-miR-20b-5p PTGFRN 1 1 1 3

hsa-miR-107 LCOR 1 1 1 3

hsa-miR-363-3p FNIP1 1 1 1 3

hsa-miR-107 PLEKHF2 1 1 1 3

hsa-miR-24-3p MEN1 1 1 1 3

hsa-miR-20b-5p NAGK 1 1 1 3

hsa-miR-17-5p ZNF107 1 1 1 3

hsa-miR-10a-5p H3F3B 1 1 1 3

hsa-miR-17-5p SKIL 1 1 1 3

hsa-miR-27a-3p NFE2L2 1 1 1 3

hsa-miR-216b-5p PPP2CB 1 1 1 3

hsa-miR-22-3p LIN7C 1 1 1 3

hsa-miR-20b-5p ZNF800 1 1 1 3

hsa-miR-23b-3p RRAS2 1 1 1 3

hsa-miR-20b-5p CIT 1 1 1 3

hsa-miR-125b-5p TP53INP1 1 1 1 3

hsa-miR-129-5p SLBP 1 1 1 3

hsa-miR-20b-5p AKTIP 1 1 1 3

hsa-miR-20b-5p PANK3 1 1 1 3

hsa-miR-17-5p SERF1A 1 1 1 3

hsa-miR-206 NUP50 1 1 1 3

hsa-miR-125b-5p RYBP 1 1 1 3

hsa-miR-107 GPCPD1 1 1 1 3

hsa-miR-17-5p CREB1 1 1 1 3

hsa-miR-363-3p MED19 1 1 1 3

hsa-miR-23b-3p CELF1 1 1 1 3

hsa-miR-17-5p MAP3K12 1 1 1 3

hsa-miR-363-3p C11orf24 1 1 1 3

hsa-miR-363-3p ERGIC2 1 1 1 3

hsa-miR-20b-5p KIF23 1 1 1 3

hsa-miR-363-3p SLC12A5 1 1 1 3

hsa-miR-17-5p TMEM245 1 1 1 3

hsa-miR-23b-3p UQCRFS1 1 1 1 3

hsa-miR-17-5p NACC2 1 1 1 3

hsa-miR-20b-5p LIMK1 1 1 1 3

hsa-miR-301b-3p PXK 1 1 1 3

hsa-miR-761 TMEM248 1 1 1 3

hsa-miR-107 AGO1 1 1 1 3

hsa-miR-17-5p TSG101 1 1 1 3

hsa-miR-20b-5p TNFAIP1 1 1 1 3

hsa-miR-107 PAFAH1B2 1 1 1 3

hsa-miR-301b-3p IER3IP1 1 1 1 3

hsa-miR-17-5p SOX4 1 1 1 3

hsa-miR-27a-3p SOS1 1 1 1 3

hsa-miR-301b-3p VPS37A 1 1 1 3

hsa-miR-140-5p GLRX5 1 1 1 3

hsa-miR-20b-5p SACS 1 1 1 3

hsa-miR-761 VAV2 1 1 1 3

hsa-miR-20b-5p MTF1 1 1 1 3

hsa-miR-1297 CREBRF 1 1 1 3

hsa-miR-301b-3p UBN2 1 1 1 3

hsa-miR-107 AXIN2 1 1 1 3

hsa-miR-301b-3p TGFBR2 1 1 1 3

hsa-miR-17-5p BBX 1 1 1 3

hsa-miR-27a-3p PAIP2 1 1 1 3

hsa-miR-363-3p DNAJB9 1 1 1 3

hsa-miR-20b-5p RLIM 1 1 1 3

hsa-miR-125b-5p CSNK2A1 1 1 1 3

hsa-miR-20b-5p ELK4 1 1 1 3

hsa-miR-363-3p S1PR1 1 1 1 3

hsa-miR-17-5p SIKE1 1 1 1 3

hsa-miR-125b-5p BTG2 1 1 1 3

hsa-miR-20b-5p SCAMP2 1 1 1 3

hsa-miR-455-5p LYPD3 1 1 1 3

hsa-miR-24-3p ADD1 1 1 1 3

hsa-miR-17-5p UBE2Q2 1 1 1 3

hsa-miR-125a-5p SH3BP5L 1 1 1 3

hsa-miR-363-3p MYLIP 1 1 1 3

hsa-miR-17-5p SAMD12 1 1 1 3

hsa-miR-363-3p GOLGA3 1 1 1 3

hsa-miR-125b-5p CGN 1 1 1 3

hsa-miR-17-5p ZNFX1 1 1 1 3

hsa-miR-140-5p FZD6 1 1 1 3

hsa-miR-22-3p NET1 1 1 1 3

hsa-miR-301b-3p HBP1 1 1 1 3

hsa-miR-363-3p CIC 1 1 1 3

hsa-miR-17-5p TP53INP1 1 1 1 3

hsa-miR-125a-5p LIN28A 1 1 1 3

hsa-miR-3619-5p HDGF 1 1 1 3

hsa-miR-17-5p ZC3H12C 1 1 1 3

hsa-miR-107 YWHAH 1 1 1 3

hsa-miR-20b-5p ATAD2 1 1 1 3

hsa-miR-3619-5p ZBTB10 1 1 1 3

hsa-miR-125b-5p ANKRD33B 1 1 1 3

hsa-miR-20b-5p KIAA0922 1 1 1 3

hsa-miR-363-3p CCSER2 1 1 1 3

hsa-miR-20b-5p PPP6R3 1 1 1 3

hsa-miR-20b-5p HAS2 1 1 1 3

hsa-miR-17-5p SMOC1 1 1 1 3

hsa-miR-20b-5p MAP3K3 1 1 1 3

hsa-miR-125b-5p BMPR1B 1 1 1 3

hsa-miR-20b-5p ELAVL2 1 1 1 3

hsa-miR-17-5p ZFYVE26 1 1 1 3

hsa-miR-17-5p RAB22A 1 1 1 3

hsa-miR-17-5p HAUS8 1 1 1 3

hsa-miR-20b-5p SEMA4B 1 1 1 3

hsa-miR-17-5p ZBTB7A 1 1 1 3

hsa-miR-301b-3p PPP6R1 1 1 1 3

hsa-miR-301b-3p EDN1 1 1 1 3

hsa-miR-22-3p YWHAZ 1 1 1 3

hsa-miR-425-5p LCOR 1 1 1 3

hsa-miR-140-5p RALA 1 1 1 3

hsa-miR-27a-3p KMT2C 1 1 1 3

hsa-miR-10a-5p BCR 1 1 1 3

hsa-miR-24-3p PER2 1 1 1 3

hsa-miR-17-5p PPP6R3 1 1 1 3

hsa-miR-27a-3p TMEM167A 1 1 1 3

hsa-miR-129-5p SESN3 1 1 1 3

hsa-miR-107 PCSK5 1 1 1 3

hsa-miR-17-5p GOLGA1 1 1 1 3

hsa-miR-17-5p TFAM 1 1 1 3

hsa-miR-23b-3p NLGN4X 1 1 1 3

hsa-miR-206 RNF138 1 1 1 3

hsa-miR-3619-5p NAP1L4 1 1 1 3

hsa-miR-338-3p ORC4 1 1 1 3

hsa-miR-1297 TNRC6B 1 1 1 3

hsa-miR-17-5p FEM1C 1 1 1 3

hsa-miR-1297 SACS 1 1 1 3

hsa-miR-17-5p UBE3C 1 1 1 3

hsa-miR-17-5p ULK1 1 1 1 3

hsa-miR-23b-3p C8orf58 1 1 1 3

hsa-miR-20b-5p ZBTB4 1 1 1 3

hsa-miR-125b-5p RPS6KA1 1 1 1 3

hsa-miR-20b-5p UNK 1 1 1 3

hsa-miR-125a-5p CSNK2A1 1 1 1 3

hsa-miR-363-3p ANP32E 1 1 1 3

hsa-miR-301b-3p NIPA1 1 1 1 3

hsa-miR-20b-5p SHOC2 1 1 1 3

hsa-miR-27a-3p HNRNPF 1 1 1 3

hsa-miR-20b-5p PLS1 1 1 1 3

hsa-miR-20b-5p FAM210A 1 1 1 3

hsa-miR-125a-5p BTG2 1 1 1 3

hsa-miR-363-3p KLHL15 1 1 1 3

hsa-miR-107 PHKA1 1 1 1 3

hsa-miR-24-3p AMOTL2 1 1 1 3

hsa-miR-22-3p SP1 1 1 1 3

hsa-miR-27a-3p SZRD1 1 1 1 3

hsa-miR-129-5p RNF165 1 1 1 3

hsa-miR-129-5p ACSL4 1 1 1 3

hsa-miR-20b-5p TWF1 1 1 1 3

hsa-miR-24-3p MAP3K9 1 1 1 3

hsa-miR-23b-3p RBPMS2 1 1 1 3

hsa-miR-363-3p LHFPL2 1 1 1 3

hsa-miR-140-5p VEGFA 1 1 1 3

hsa-miR-363-3p PAPD7 1 1 1 3

hsa-miR-20b-5p SQSTM1 1 1 1 3

hsa-miR-24-3p TAOK1 1 1 1 3

hsa-miR-17-5p KIAA0922 1 1 1 3

hsa-miR-129-5p CBX4 1 1 1 3

hsa-miR-507 DNAJC15 1 1 1 3

hsa-miR-17-5p SESN3 1 1 1 3

hsa-miR-17-5p TBC1D2 1 1 1 3

hsa-miR-23b-3p TMED7 1 1 1 3

hsa-miR-17-5p CAMTA1 1 1 1 3

hsa-miR-107 ARIH1 1 1 1 3

hsa-miR-3619-5p CDC42SE1 1 1 1 3

hsa-miR-24-3p ZXDA 1 1 1 3

hsa-miR-3619-5p TBPL1 1 1 1 3

hsa-miR-3619-5p MFN2 1 1 1 3

hsa-miR-23b-3p PPP1CB 1 1 1 3

hsa-miR-125b-5p ABTB1 1 1 1 3

hsa-miR-125a-5p TP53INP1 1 1 1 3

hsa-miR-146b-5p TRAF6 1 1 1 3

hsa-miR-27a-3p RPS6KA5 1 1 1 3

hsa-miR-363-3p ZFYVE21 1 1 1 3

hsa-miR-206 BSCL2 1 1 1 3

hsa-miR-10a-5p RAP2A 1 1 1 3

hsa-miR-20b-5p ZBTB18 1 1 1 3

hsa-miR-17-5p KLHL20 1 1 1 3

hsa-miR-206 GPD2 1 1 1 3

hsa-miR-17-5p PGM2L1 1 1 1 3

hsa-miR-301b-3p CDADC1 1 1 1 3

hsa-miR-17-5p CCSER2 1 1 1 3

hsa-miR-20b-5p ANKRD12 1 1 1 3

hsa-miR-20b-5p E2F5 1 1 1 3

hsa-miR-17-5p PXK 1 1 1 3

hsa-miR-20b-5p CLOCK 1 1 1 3

hsa-miR-17-5p TNKS2 1 1 1 3

hsa-miR-24-3p VGLL3 1 1 1 3

hsa-miR-17-5p ZBTB18 1 1 1 3

hsa-miR-20b-5p ARHGAP12 1 1 1 3

hsa-miR-140-5p RAB10 1 1 1 3

hsa-miR-17-5p PKD2 1 1 1 3

hsa-miR-17-5p C9orf40 1 1 1 3

hsa-miR-301b-3p MBNL3 1 1 1 3

hsa-miR-24-3p GBA2 1 1 1 3

hsa-miR-17-5p STK11 1 1 1 3

hsa-miR-107 VPS4A 1 1 1 3

hsa-miR-17-5p BMPR2 1 1 1 3

hsa-miR-301b-3p SALL3 1 1 1 3

hsa-miR-216b-5p DNAJB9 1 1 1 3

hsa-miR-17-5p BRMS1L 1 1 1 3

hsa-miR-107 PRKCE 1 1 1 3

hsa-miR-363-3p BAZ2B 1 1 1 3

hsa-miR-193a-3p E2F6 1 1 1 3

hsa-miR-17-5p AKAP11 1 1 1 3

hsa-miR-27a-3p CD2AP 1 1 1 3

hsa-miR-17-5p PIP4K2C 1 1 1 3

hsa-miR-876-3p MCL1 1 1 1 3

hsa-miR-27a-3p ENDOU 1 1 1 3

hsa-miR-125a-5p ANKRD33B 1 1 1 3

hsa-miR-27a-3p RREB1 1 1 1 3

hsa-miR-107 IGSF3 1 1 1 3

hsa-miR-425-5p ARIH1 1 1 1 3

hsa-miR-17-5p CEP97 1 1 1 3

hsa-miR-129-5p DNAJC15 1 1 1 3

hsa-miR-17-5p NKIRAS1 1 1 1 3

hsa-miR-140-5p STRADB 1 1 1 3

hsa-miR-27a-3p SEC24A 1 1 1 3

hsa-miR-107 BTLA 1 1 1 3

hsa-miR-125a-5p MTUS1 1 1 1 3

hsa-miR-125b-5p SH3BP5L 1 1 1 3

hsa-miR-129-5p SEMA6A 1 1 1 3

hsa-miR-363-3p FASLG 1 1 1 3

hsa-miR-23b-3p SEMA6D 1 1 1 3

hsa-miR-20b-5p RAB10 1 1 1 3

hsa-miR-20b-5p NFAT5 1 1 1 3

hsa-miR-1297 KPNA2 1 1 1 3

hsa-miR-125b-5p LIN28A 1 1 1 3

hsa-miR-125b-5p CCNJ 1 1 1 3

hsa-miR-20b-5p LIMA1 1 1 1 3

hsa-miR-129-5p FNIP2 1 1 1 3

hsa-miR-17-5p TXNIP 1 1 1 3

hsa-miR-3619-5p ERC1 1 1 1 3

hsa-miR-20b-5p ACSL4 1 1 1 3

hsa-miR-24-3p MATR3 1 1 1 3

hsa-miR-17-5p SCAMP2 1 1 1 3

hsa-miR-17-5p CCDC71L 1 1 1 3

hsa-miR-22-3p H3F3B 1 1 1 3

hsa-miR-27a-3p HOXC6 1 1 1 3

hsa-miR-1297 WNK1 1 1 1 3

hsa-miR-507 BCL7A 1 1 1 3

hsa-miR-22-3p TET2 1 1 1 3

hsa-miR-17-5p ITGB8 1 1 1 3

hsa-miR-301b-3p RFX7 1 1 1 3

hsa-miR-20b-5p ARID4B 1 1 1 3

hsa-miR-129-5p EI24 1 1 1 3

hsa-miR-876-3p PPIA 1 1 1 3

hsa-miR-125b-5p SLC35A4 1 1 1 3

hsa-miR-363-3p MAP2K4 1 1 1 3

hsa-miR-17-5p OXR1 1 1 1 3

hsa-miR-107 RUNX1T1 1 1 1 3

hsa-miR-20b-5p SCAMP5 1 1 1 3

hsa-miR-20b-5p TMEM123 1 1 1 3

hsa-miR-27a-3p ABCA1 1 1 1 3

hsa-miR-17-5p SEMA7A 1 1 1 3

hsa-miR-301b-3p JARID2 1 1 1 3

hsa-miR-17-5p CAPRIN2 1 1 1 3

hsa-miR-613 NR1H3 1 1 1 3

hsa-miR-107 N4BP1 1 1 1 3

hsa-miR-301b-3p EOGT 1 1 1 3

hsa-miR-17-5p CLIP4 1 1 1 3

hsa-miR-17-5p FYCO1 1 1 1 3

hsa-miR-27a-3p CCNK 1 1 1 3

hsa-miR-10a-5p EPHA4 1 1 1 3

hsa-miR-17-5p TMEM242 1 1 1 3

hsa-miR-129-5p KANK4 1 1 1 3

hsa-miR-17-5p ADARB1 1 1 1 3

hsa-miR-10a-5p TRIM2 1 1 1 3

hsa-miR-17-5p DNAJB9 1 1 1 3

hsa-miR-193a-3p TMEM30A 1 1 1 3

hsa-miR-10a-5p GALNT1 1 1 1 3

hsa-miR-107 DHX33 1 1 1 3

hsa-miR-125a-5p ESRRA 1 1 1 3

hsa-miR-20b-5p FBXO21 1 1 1 3

hsa-miR-20b-5p RAP2C 1 1 1 3

hsa-miR-23b-3p PTEN 1 1 1 3

hsa-miR-24-3p RAP2C 1 1 1 3

hsa-miR-140-5p TSC22D2 1 1 1 3

hsa-miR-507 PTPRM 1 1 1 3

hsa-miR-20b-5p LPGAT1 1 1 1 3

hsa-miR-17-5p MCC 1 1 1 3

hsa-miR-17-5p KLF10 1 1 1 3

hsa-miR-125b-5p KCNS3 1 1 1 3

hsa-miR-129-5p CLOCK 1 1 1 3

hsa-miR-107 CPEB3 1 1 1 3

hsa-miR-301b-3p HPRT1 1 1 1 3

hsa-miR-301b-3p MSMO1 1 1 1 3

hsa-miR-125a-5p SGPL1 1 1 1 3

hsa-miR-27a-3p MKNK2 1 1 1 3

hsa-miR-425-5p SLC16A1 1 1 1 3

hsa-miR-107 SALL1 1 1 1 3

hsa-miR-1297 ALG1 1 1 1 3

hsa-miR-20b-5p UBE2Q2 1 1 1 3

hsa-miR-17-5p TMEM64 1 1 1 3

hsa-miR-20b-5p TADA2B 1 1 1 3

hsa-miR-20b-5p GIGYF1 1 1 1 3

hsa-miR-17-5p FBXO31 1 1 1 3

hsa-miR-22-3p CSF1R 1 1 1 3

hsa-miR-490-3p CLCC1 1 1 1 3

hsa-miR-129-5p KAT6B 1 1 1 3

hsa-miR-20b-5p EGLN3 1 1 1 3

hsa-miR-129-5p CALM1 1 1 1 3

hsa-miR-17-5p DNAJC27 1 1 1 3

hsa-miR-3619-5p TRIM29 1 1 1 3

hsa-miR-17-5p MAP3K3 1 1 1 3

hsa-miR-363-3p GPBP1L1 1 1 1 3

hsa-miR-17-5p SLC16A9 1 1 1 3

hsa-miR-20b-5p RUNDC1 1 1 1 3

hsa-miR-363-3p CCDC113 1 1 1 3

hsa-miR-24-3p TRPM6 1 1 1 3

hsa-miR-206 WEE1 1 1 1 3

hsa-miR-17-5p TRIP10 1 1 1 3

hsa-miR-17-5p WDR37 1 1 1 3

hsa-miR-107 ITGA2 1 1 1 3

hsa-miR-20b-5p PPP1R3B 1 1 1 3

hsa-miR-17-5p CEP170 1 1 1 3

hsa-miR-206 BDNF 1 1 1 3

hsa-miR-20b-5p VPS26A 1 1 1 3

hsa-miR-27a-3p PPIF 1 1 1 3

hsa-miR-1297 EDEM3 1 1 1 3

hsa-miR-129-5p ETV6 1 1 1 3

hsa-miR-17-5p USP28 1 1 1 3

hsa-miR-17-5p SACS 1 1 1 3

hsa-miR-363-3p KIF5B 1 1 1 3

hsa-miR-27a-3p FOXP4 1 1 1 3

hsa-miR-27a-3p LYSMD3 1 1 1 3

hsa-miR-507 UNKL 1 1 1 3

hsa-miR-17-5p CERCAM 1 1 1 3

hsa-miR-761 AHNAK2 1 1 1 3

hsa-miR-301b-3p TROVE2 1 1 1 3

hsa-miR-17-5p ZNF202 1 1 1 3

hsa-miR-125a-5p PCTP 1 1 1 3

hsa-miR-363-3p MAP1B 1 1 1 3

hsa-miR-17-5p KMT2A 1 1 1 3

hsa-miR-23b-3p ATXN7L3B 1 1 1 3

hsa-miR-24-3p FURIN 1 1 1 3

hsa-miR-10a-5p MTF2 1 1 1 3

hsa-miR-301b-3p TRIM37 1 1 1 3

hsa-miR-17-5p TRIM37 1 1 1 3

hsa-miR-20b-5p KATNAL1 1 1 1 3

hsa-miR-27a-3p TMEM170B 1 1 1 3

hsa-miR-24-3p POGZ 1 1 1 3

hsa-miR-20b-5p BICD2 1 1 1 3

hsa-miR-107 FBXW7 1 1 1 3

hsa-miR-17-5p TMBIM6 1 1 1 3

hsa-miR-22-3p MAX 1 1 1 3

hsa-miR-10a-5p CRLF3 1 1 1 3

hsa-miR-107 KIF23 1 1 1 3

hsa-miR-1297 UBE2H 1 1 1 3

hsa-miR-301b-3p RBM20 1 1 1 3

hsa-miR-17-5p AKTIP 1 1 1 3

hsa-miR-20b-5p PPP3R1 1 1 1 3

hsa-miR-20b-5p TMEM127 1 1 1 3

hsa-miR-20b-5p FOXJ2 1 1 1 3

hsa-miR-507 ZBTB5 1 1 1 3

hsa-miR-193a-3p KMT2A 1 1 1 3

hsa-miR-301b-3p FZD6 1 1 1 3

hsa-miR-17-5p EIF5A2 1 1 1 3

hsa-miR-193a-3p ABI2 1 1 1 3

hsa-miR-363-3p MOAP1 1 1 1 3

hsa-miR-20b-5p ZNFX1 1 1 1 3

hsa-miR-23b-3p CNN2 1 1 1 3

hsa-miR-27a-3p ZFP36L1 1 1 1 3

hsa-miR-17-5p KATNAL1 1 1 1 3

hsa-miR-20b-5p UBXN2A 1 1 1 3

hsa-miR-20b-5p SLK 1 1 1 3

hsa-miR-23b-3p TNFAIP3 1 1 1 3

hsa-miR-24-3p POLR3D 1 1 1 3

hsa-miR-20b-5p PHTF2 1 1 1 3

hsa-miR-17-5p CCND1 1 1 1 3

hsa-miR-24-3p FSCN1 1 1 1 3

hsa-miR-20b-5p VPS13C 1 1 1 3

hsa-miR-27a-3p LIFR 1 1 1 3

hsa-miR-17-5p HMGB3 1 1 1 3

hsa-miR-20b-5p ATL3 1 1 1 3

hsa-miR-20b-5p GNS 1 1 1 3

hsa-miR-24-3p CMTM4 1 1 1 3

hsa-miR-20b-5p SALL3 1 1 1 3

hsa-miR-27a-3p ABL2 1 1 1 3

hsa-miR-17-5p ARID4B 1 1 1 3

hsa-miR-17-5p SUCO 1 1 1 3

hsa-miR-22-3p BTG1 1 1 1 3

hsa-miR-761 KCTD15 1 1 1 3

hsa-miR-20b-5p NRIP3 1 1 1 3

hsa-miR-17-5p FBXO48 1 1 1 3

hsa-miR-140-5p ARIH1 1 1 1 3

hsa-miR-17-5p NABP1 1 1 1 3

hsa-miR-17-5p FAM210A 1 1 1 3

hsa-miR-17-5p PPP1R3B 1 1 1 3

hsa-miR-27a-3p PDHX 1 1 1 3

hsa-miR-24-3p DNAJB12 1 1 1 3

hsa-miR-129-5p HBS1L 1 1 1 3

hsa-miR-24-3p FGFR3 1 1 1 3

hsa-miR-125b-5p PCTP 1 1 1 3

hsa-miR-23b-3p MCFD2 1 1 1 3

hsa-miR-17-5p SOCS6 1 1 1 3

hsa-miR-17-5p PIP4K2A 1 1 1 3

hsa-miR-24-3p C1orf106 1 1 1 3

hsa-miR-20b-5p MCL1 1 1 1 3

hsa-miR-301b-3p ENPP5 1 1 1 3

hsa-miR-20b-5p DPYSL2 1 1 1 3

hsa-miR-27a-3p NEURL1B 1 1 1 3

hsa-miR-613 FNDC3A 1 1 1 3

hsa-miR-24-3p CNNM3 1 1 1 3

hsa-miR-20b-5p FAM129A 1 1 1 3

hsa-miR-17-5p ZNF417 1 1 1 3

hsa-miR-24-3p RNF11 1 1 1 3

hsa-miR-206 HSP90B1 1 1 1 3

hsa-miR-24-3p H2AFX 1 1 1 3

hsa-miR-23b-3p PRR14L 1 1 1 3

hsa-miR-107 SALL4 1 1 1 3

hsa-miR-17-5p RAB10 1 1 1 3

hsa-miR-17-5p STK17B 1 1 1 3

hsa-miR-10a-5p HOXA1 1 1 1 3

hsa-miR-338-3p ARHGEF28 1 1 1 3

hsa-miR-23b-3p SDHD 1 1 1 3

hsa-miR-125b-5p SGPL1 1 1 1 3

hsa-miR-17-5p FCHO2 1 1 1 3

hsa-miR-107 PAWR 1 1 1 3

hsa-miR-206 KCNJ2 1 1 1 3

hsa-miR-20b-5p SESN3 1 1 1 3

hsa-miR-20b-5p ZC3H12C 1 1 1 3

hsa-miR-10a-5p TIAM1 1 1 1 3

hsa-miR-10a-5p CHL1 1 1 1 3

hsa-miR-20b-5p REEP3 1 1 1 3

hsa-miR-23b-3p TAB3 1 1 1 3

hsa-miR-27a-3p H3F3B 1 1 1 3

hsa-miR-27a-3p TROVE2 1 1 1 3

hsa-miR-17-5p ERAP1 1 1 1 3

hsa-miR-17-5p OSTM1 1 1 1 3

hsa-miR-23b-3p BTLA 1 1 1 3

hsa-miR-20b-5p MTMR3 1 1 1 3

hsa-miR-17-5p SMAD5 1 1 1 3

hsa-miR-24-3p AVL9 1 1 1 3

hsa-miR-761 CS 1 1 1 3

hsa-miR-20b-5p ARHGAP1 1 1 1 3

hsa-miR-20b-5p NABP1 1 1 1 3

hsa-miR-17-5p PTP4A1 1 1 1 3

hsa-miR-107 RIMS3 1 1 1 3

hsa-miR-20b-5p SPOPL 1 1 1 3

hsa-miR-20b-5p FBXO31 1 1 1 3

hsa-miR-17-5p MINK1 1 1 1 3

hsa-miR-125a-5p SIRT7 1 1 1 3

hsa-miR-1244 CDK17 1 1 1 3

hsa-miR-3619-5p FLOT2 1 1 1 3

hsa-miR-363-3p WASL 1 1 1 3

hsa-miR-17-5p CEP57 1 1 1 3

hsa-miR-363-3p DDX3X 1 1 1 3

hsa-miR-22-3p EFR3B 1 1 1 3

hsa-miR-20b-5p SMOC1 1 1 1 3

hsa-miR-216b-5p TPM3 1 1 1 3

hsa-miR-27a-3p GSE1 1 1 1 3

hsa-miR-17-5p ATL3 1 1 1 3

hsa-miR-125b-5p SLC7A6 1 1 1 3

hsa-miR-17-5p PITPNA 1 1 1 3

hsa-miR-107 MCM7 1 1 1 3

hsa-miR-22-3p LRRC1 1 1 1 3

hsa-miR-125b-5p PPAT 1 1 1 3

hsa-miR-1297 SLC25A36 1 1 1 3

hsa-miR-22-3p RCC2 1 1 1 3

hsa-miR-1297 LOXL2 1 1 1 3

hsa-miR-17-5p FAM57A 1 1 1 3

hsa-miR-193a-3p PLAU 1 1 1 3

hsa-miR-27a-3p ADORA2B 1 1 1 3

hsa-miR-363-3p RRN3 1 1 1 3

hsa-miR-24-3p SESN1 1 1 1 3

hsa-miR-20b-5p ABHD2 1 1 1 3

hsa-miR-20b-5p EFCAB14 1 1 1 3

hsa-miR-3619-5p VAV2 1 1 1 3

hsa-miR-761 PIM1 1 1 1 3

hsa-miR-20b-5p RAB5B 1 1 1 3

hsa-miR-301b-3p CDK19 1 1 1 3

hsa-miR-140-5p KLK10 1 1 1 3

hsa-miR-20b-5p C7orf43 1 1 1 3

hsa-miR-216b-5p MCM4 1 1 1 3

hsa-miR-301b-3p PRUNE2 1 1 1 3

hsa-miR-27a-3p PHLPP2 1 1 1 3

hsa-miR-17-5p MAP3K2 1 1 1 3

hsa-miR-363-3p SOX4 1 1 1 3

hsa-miR-20b-5p SLC22A23 1 1 1 3

hsa-miR-24-3p TOR2A 1 1 1 3

hsa-miR-17-5p MAP3K9 1 1 1 3

hsa-miR-301b-3p RACGAP1 1 1 1 3

hsa-miR-761 PNPLA6 1 1 1 3

hsa-miR-761 HDGF 1 1 1 3

hsa-miR-425-5p MAP2K6 1 1 1 3

hsa-miR-17-5p FRMD6 1 1 1 3

hsa-miR-125a-5p ATXN1 1 1 1 3

hsa-miR-17-5p FOXQ1 1 1 1 3

hsa-miR-301b-3p SPG20 1 1 1 3

hsa-miR-129-5p CBX6 1 1 1 3

hsa-miR-125b-5p PRDM1 1 1 1 3

hsa-miR-20b-5p STAT3 1 1 1 3

hsa-miR-17-5p CNOT7 1 1 1 3

hsa-miR-23b-3p FNIP1 1 1 1 3

hsa-miR-27a-3p SLC7A11 1 1 1 3

hsa-miR-17-5p NRIP3 1 1 1 3

hsa-miR-206 CERS2 1 1 1 3

hsa-miR-301b-3p USP13 1 1 1 3

hsa-miR-20b-5p FAM126B 1 1 1 3

hsa-miR-27a-3p UBE2D1 1 1 1 3

hsa-miR-17-5p DPYSL2 1 1 1 3

hsa-miR-20b-5p CERCAM 1 1 1 3

hsa-miR-20b-5p ARAP2 1 1 1 3

hsa-miR-17-5p RORA 1 1 1 3

hsa-miR-17-5p NAPEPLD 1 1 1 3

hsa-miR-301b-3p GMFB 1 1 1 3

hsa-miR-363-3p GNAQ 1 1 1 3

hsa-miR-17-5p NUP35 1 1 1 3

hsa-miR-20b-5p PITPNA 1 1 1 3

hsa-miR-20b-5p CEP57 1 1 1 3

hsa-miR-24-3p ZXDB 1 1 1 3

hsa-miR-17-5p EGLN3 1 1 1 3

hsa-miR-107 ZBTB10 1 1 1 3

hsa-miR-17-5p E2F5 1 1 1 3

hsa-miR-24-3p CD34 1 1 1 3

hsa-miR-301b-3p MBNL1 1 1 1 3

hsa-miR-107 ZNF606 1 1 1 3

hsa-miR-363-3p PLEKHA1 1 1 1 3

hsa-miR-20b-5p FYCO1 1 1 1 3

hsa-miR-22-3p CHD9 1 1 1 3

hsa-miR-27a-3p ELL2 1 1 1 3

hsa-miR-17-5p SALL3 1 1 1 3

hsa-miR-17-5p PHF6 1 1 1 3

hsa-miR-125b-5p ZNF385A 1 1 1 3

hsa-miR-17-5p SGTB 1 1 1 3

hsa-miR-27a-3p APPBP2 1 1 1 3

hsa-miR-27a-3p AKIRIN1 1 1 1 3

hsa-miR-22-3p FRAT2 1 1 1 3

hsa-miR-301b-3p CAMSAP2 1 1 1 3

hsa-miR-1297 TBC1D13 1 1 1 3

hsa-miR-17-5p ARAP2 1 1 1 3

hsa-miR-20b-5p KIAA1147 1 1 1 3

hsa-miR-27a-3p MBTD1 1 1 1 3

hsa-miR-20b-5p NIN 1 1 1 3

hsa-miR-301b-3p CEP55 1 1 1 3

hsa-miR-3619-5p JAG2 1 1 1 3

hsa-miR-17-5p CHAF1A 1 1 1 3

hsa-miR-363-3p GID4 1 1 1 3

hsa-miR-216b-5p ARL6IP1 1 1 1 3

hsa-miR-10a-5p RORA 1 1 1 3

hsa-miR-20b-5p ZFYVE26 1 1 1 3

hsa-miR-17-5p EIF4H 1 1 1 3

hsa-miR-27a-3p FBXW7 1 1 1 3

hsa-miR-27a-3p SEMA7A 1 1 1 3

hsa-miR-107 SOWAHC 1 1 1 3

hsa-miR-27a-3p MED13 1 1 1 3

hsa-miR-301b-3p BTBD3 1 1 1 3

hsa-miR-27a-3p PLAGL2 1 1 1 3

hsa-miR-17-5p KIAA1191 1 1 1 3

hsa-miR-20b-5p LDLR 1 1 1 3

hsa-miR-3619-5p C10orf76 1 1 1 3

hsa-miR-17-5p SLC22A23 1 1 1 3

hsa-miR-876-3p OR9Q1 1 1 1 3

hsa-miR-20b-5p ORMDL3 1 1 1 3

hsa-miR-23b-3p MET 1 1 1 3

hsa-miR-363-3p COX20 1 1 1 3

hsa-miR-17-5p PDLIM5 1 1 1 3

hsa-miR-425-5p BEX4 1 1 1 3

hsa-miR-107 UBR3 1 1 1 3

hsa-miR-22-3p KCTD10 1 1 1 3

hsa-miR-20b-5p NRBP1 1 1 1 3

hsa-miR-193a-3p WDR82 1 1 1 3

hsa-miR-17-5p RRM2 1 1 1 3

hsa-miR-125b-5p SIRT7 1 1 1 3

hsa-miR-24-3p INSIG1 1 1 1 3

hsa-miR-3619-5p CRKL 1 1 1 3

hsa-miR-1297 NABP1 1 1 1 3

hsa-miR-129-5p RSBN1 1 1 1 3

hsa-miR-20b-5p RAB22A 1 1 1 3

hsa-miR-17-5p KMT2B 1 1 1 3

hsa-miR-20b-5p RUFY2 1 1 1 3

hsa-miR-27a-3p RMND5A 1 1 1 3

hsa-miR-17-5p REEP3 1 1 1 3

hsa-miR-107 HIC2 1 1 1 3

hsa-miR-301b-3p ACSL4 1 1 1 3

hsa-miR-27a-3p TRIM23 1 1 1 3

hsa-miR-338-3p ZWINT 1 1 1 3

hsa-miR-17-5p RAPGEF4 1 1 1 3

hsa-miR-20b-5p PPP6C 1 1 1 3

hsa-miR-17-5p ANKRD50 1 1 1 3

hsa-miR-761 ZNRF1 1 1 1 3

hsa-miR-10a-5p NCOR2 1 1 1 3

hsa-miR-125a-5p ZNF385A 1 1 1 3

hsa-miR-17-5p FBXL5 1 1 1 3

hsa-miR-20b-5p FOXJ3 1 1 1 3

hsa-miR-24-3p IFNG 1 1 1 3

hsa-miR-363-3p UBE2Z 1 1 1 3

hsa-miR-20b-5p DDX5 1 1 1 3

hsa-miR-20b-5p CFL2 1 1 1 3

hsa-miR-17-5p BMP8B 1 1 1 3

hsa-miR-17-5p BTBD7 1 1 1 3

hsa-miR-107 ASH1L 1 1 1 3

hsa-miR-425-5p PNMAL1 1 1 1 3

hsa-miR-301b-3p SOX4 1 1 1 3

hsa-miR-27a-3p PLAG1 1 1 1 3

hsa-miR-20b-5p AGO1 1 1 1 3

hsa-miR-301b-3p MAPK1 1 1 1 3

hsa-miR-24-3p MBD6 1 1 1 3

hsa-miR-17-5p MKNK2 1 1 1 3

hsa-miR-27a-3p DCUN1D4 1 1 1 3

hsa-miR-125b-5p MTF1 1 1 1 3

hsa-miR-363-3p KLHDC10 1 1 1 3

hsa-miR-301b-3p USP32 1 1 1 3

hsa-miR-22-3p RCOR1 1 1 1 3

hsa-miR-140-5p 2-Sep 1 1 1 3

hsa-miR-876-3p ARID1A 1 1 1 3

hsa-miR-3619-5p TMED9 1 1 1 3

hsa-miR-20b-5p FRMD6 1 1 1 3

hsa-miR-107 RAB10 1 1 1 3

hsa-miR-129-5p KLHL5 1 1 1 3

hsa-miR-3619-5p AHNAK2 1 1 1 3

hsa-miR-27a-3p PHB 1 1 1 3

hsa-miR-107 SLAIN2 1 1 1 3

hsa-miR-17-5p SQSTM1 1 1 1 3

hsa-miR-20b-5p PTPN4 1 1 1 3

hsa-miR-24-3p DVL3 1 1 1 3

hsa-miR-1297 MAPK6 1 1 1 3

hsa-miR-125a-5p PRDM1 1 1 1 3

hsa-miR-20b-5p 2-Sep 1 1 1 3

hsa-miR-140-5p HDAC7 1 1 1 3

hsa-miR-129-5p APC 1 1 1 3

hsa-miR-363-3p PER2 1 1 1 3

hsa-miR-17-5p EFCAB14 1 1 1 3

hsa-miR-20b-5p MINK1 1 1 1 3

hsa-miR-20b-5p HMGB3 1 1 1 3

hsa-miR-107 SUN2 1 1 1 3

hsa-miR-24-3p MXI1 1 1 1 3

hsa-miR-107 SNCG 1 1 1 3

hsa-miR-17-5p ARHGAP35 1 1 1 3

hsa-miR-17-5p LYSMD3 1 1 1 3

hsa-miR-761 FAM49B 1 1 1 3

hsa-miR-27a-3p FOXO1 1 1 1 3

hsa-miR-140-5p LAMC1 1 1 1 3

hsa-miR-20b-5p REST 1 1 1 3

hsa-miR-17-5p RPF2 1 1 1 3

hsa-miR-17-5p NETO2 1 1 1 3

hsa-miR-125b-5p ATXN1 1 1 1 3

hsa-miR-129-5p GALNT1 1 1 1 3

hsa-miR-455-5p DDX3X 1 1 1 3

hsa-miR-20b-5p KLHL28 1 1 1 3

hsa-miR-27a-3p RNF182 1 1 1 3

hsa-miR-27a-3p CELF2 1 1 1 3

hsa-miR-17-5p PPP6C 1 1 1 3

hsa-miR-23b-3p SOCS6 1 1 1 3

hsa-miR-3619-5p KLF16 1 1 1 3

hsa-miR-17-5p TADA2B 1 1 1 3

hsa-miR-125a-5p KLF13 1 1 1 3

hsa-miR-27a-3p DYNLL2 1 1 1 3

hsa-miR-125b-5p ARID3B 1 1 1 3

hsa-miR-301b-3p SAMD8 1 1 1 3

hsa-miR-125b-5p LIN28B 1 1 1 3

hsa-miR-363-3p TPPP 1 1 1 3

hsa-miR-363-3p INSIG1 1 1 1 3

hsa-miR-17-5p SERF1B 1 1 1 3

hsa-miR-20b-5p SAMD12 1 1 1 3

hsa-miR-24-3p CCDC58 1 1 1 3

hsa-miR-17-5p MCL1 1 1 1 3

hsa-miR-17-5p TMEM127 1 1 1 3

hsa-miR-3619-5p TMEM248 1 1 1 3

hsa-miR-301b-3p CUL3 1 1 1 3

hsa-miR-363-3p VMA21 1 1 1 3

hsa-miR-17-5p ANKRD52 1 1 1 3

hsa-miR-206 GJA1 1 1 1 3

hsa-miR-129-5p FMR1 1 1 1 3

hsa-miR-20b-5p ZBTB9 1 1 1 3

hsa-miR-301b-3p UBE2D2 1 1 1 3

hsa-miR-27a-3p NPEPPS 1 1 1 3

hsa-miR-107 ACTR2 1 1 1 3

hsa-miR-125a-5p MMP11 1 1 1 3

hsa-miR-17-5p RUFY2 1 1 1 3

hsa-miR-129-5p HMGB1 1 1 1 3

hsa-miR-193a-3p MCL1 1 1 1 3

hsa-miR-17-5p SGMS1 1 1 1 3

hsa-miR-146b-5p HNRNPD 1 1 1 3

hsa-miR-20b-5p MIDN 1 1 1 3

hsa-miR-301b-3p PIGA 1 1 1 3

hsa-miR-27a-3p FAM84B 1 1 1 3

hsa-miR-1297 FRAT2 1 1 1 3

hsa-miR-301b-3p ENPP4 1 1 1 3

hsa-miR-613 BSCL2 1 1 1 3

hsa-miR-363-3p SSFA2 1 1 1 3

hsa-miR-193a-3p INO80D 1 1 1 3

hsa-miR-3619-5p ZNF641 1 1 1 3

hsa-miR-216b-5p ZDHHC9 1 1 1 3

hsa-miR-507 BACH2 1 1 1 3

hsa-miR-129-5p SPRY4 1 1 1 3

hsa-miR-107 ELK4 1 1 1 3

hsa-miR-27a-3p LDLR 1 1 1 3

hsa-miR-17-5p CNOT4 1 1 1 3

hsa-miR-363-3p RSBN1 1 1 1 3

hsa-miR-20b-5p KLF3 1 1 1 3

hsa-miR-425-5p AFF4 1 1 1 3

hsa-miR-20b-5p SEMA7A 1 1 1 3

hsa-miR-129-5p LRRC2 1 1 1 3

hsa-miR-23b-3p GHITM 1 1 1 3

hsa-miR-17-5p NPAT 1 1 1 3

hsa-miR-193a-3p LAMC1 1 1 1 3

hsa-miR-301b-3p CHIC1 1 1 1 3

hsa-miR-17-5p RABEP1 1 1 1 3

hsa-miR-125a-5p DHX33 1 1 1 3

hsa-miR-17-5p CNOT6L 1 1 1 3

hsa-miR-363-3p SMU1 1 1 1 3

hsa-miR-17-5p RLIM 1 1 1 3

hsa-miR-17-5p LAPTM4A 1 1 1 3

hsa-miR-17-5p ZNF280B 1 1 1 3

hsa-miR-216b-5p FZD5 1 1 1 3

hsa-miR-1297 CREBZF 1 1 1 3

hsa-miR-129-5p AKAP10 1 1 1 3

hsa-miR-1297 ADM 1 1 1 3

hsa-miR-107 SEMA6A 1 1 1 3

hsa-miR-107 MTMR3 1 1 1 3

hsa-miR-23b-3p SESN2 1 1 1 3

hsa-miR-17-5p FBXO21 1 1 1 3

hsa-miR-107 CAPZA2 1 1 1 3

hsa-miR-17-5p NR2C2 1 1 1 3

hsa-miR-24-3p YRDC 1 1 1 3

hsa-miR-20b-5p BNIP2 1 1 1 3

hsa-miR-301b-3p RAB34 1 1 1 3

hsa-miR-17-5p GOLGA2 1 1 1 3

hsa-miR-17-5p STAT3 1 1 1 3

hsa-miR-20b-5p OSTM1 1 1 1 3

hsa-miR-107 POLD3 1 1 1 3

hsa-miR-301b-3p STARD13 1 1 1 3

hsa-miR-24-3p YOD1 1 1 1 3

hsa-miR-20b-5p KLHL20 1 1 1 3

hsa-miR-17-5p TWF1 1 1 1 3

hsa-miR-17-5p PPP1R15B 1 1 1 3

hsa-miR-23b-3p QSER1 1 1 1 3

hsa-miR-22-3p RGS2 1 1 1 3

hsa-miR-20b-5p TRIM37 1 1 1 3

hsa-miR-363-3p NUP43 1 1 1 3

hsa-miR-22-3p PTEN 1 1 1 3

hsa-miR-17-5p NIPA1 1 1 1 3

hsa-miR-761 AMER1 1 1 1 3

hsa-miR-140-5p MED13 1 1 1 3

hsa-miR-24-3p MLEC 1 1 1 3

hsa-miR-17-5p RAB11FIP1 1 1 1 3

hsa-miR-129-5p GNAQ 1 1 1 3

hsa-miR-363-3p MCOLN2 1 1 1 3

hsa-miR-193a-3p ARMC1 1 1 1 3

hsa-miR-20b-5p KPNA2 1 1 1 3

hsa-miR-125b-5p E2F2 1 1 1 3

hsa-miR-363-3p USP28 1 1 1 3

hsa-miR-363-3p TMF1 1 1 1 3

hsa-miR-507 JAZF1 1 1 1 3

hsa-miR-20b-5p ANKRD52 1 1 1 3

hsa-miR-17-5p ANKRD13C 1 1 1 3

hsa-miR-17-5p ATG16L1 1 1 1 3

hsa-miR-613 NUP50 1 1 1 3

hsa-miR-363-3p REV3L 1 1 1 3

hsa-miR-17-5p RBBP7 1 1 1 3

hsa-miR-363-3p BAK1 1 1 1 3

hsa-miR-17-5p FRS2 1 1 1 3

hsa-miR-761 CRKL 1 1 1 3

hsa-miR-17-5p MIDN 1 1 1 3

hsa-miR-10a-5p TFAP2C 1 1 1 3

hsa-miR-20b-5p GAB1 1 1 1 3

hsa-miR-23b-3p BRWD1 1 1 1 3

hsa-miR-507 GTF2E2 1 1 1 3

hsa-miR-17-5p RBM12B 1 1 1 3

hsa-miR-27a-3p ANKRD40 1 1 1 3

hsa-miR-363-3p TEF 1 1 1 3

hsa-miR-27a-3p CNN3 1 1 1 3

hsa-miR-20b-5p EZH1 1 1 1 3

hsa-miR-125a-5p ARID3B 1 1 1 3

hsa-miR-27a-3p THRB 1 1 1 3

hsa-miR-17-5p ZBTB4 1 1 1 3

hsa-miR-20b-5p CAPN15 1 1 1 3

hsa-miR-193a-3p KRAS 1 1 1 3

hsa-miR-125a-5p LIN28B 1 1 1 3

hsa-miR-24-3p STRADB 1 1 1 3

hsa-miR-129-5p SOX4 1 1 1 3

hsa-miR-125b-5p STARD13 1 1 1 3

hsa-miR-24-3p C17orf49 1 1 1 3

hsa-miR-20b-5p CEP170 1 1 1 3

hsa-miR-301b-3p ESR1 1 1 1 3

hsa-miR-1297 MAT2A 1 1 1 3

hsa-miR-17-5p MAPRE3 1 1 1 3

hsa-miR-22-3p BRWD3 1 1 1 3

hsa-miR-22-3p NR3C1 1 1 1 3

hsa-miR-20b-5p FEM1C 1 1 1 3

hsa-miR-17-5p IRAK4 1 1 1 3

hsa-miR-20b-5p ARHGAP35 1 1 1 3

hsa-miR-1297 SFPQ 1 1 1 3

hsa-miR-1297 MAN2A1 1 1 1 3

hsa-miR-20b-5p CHD9 1 1 1 3

hsa-miR-24-3p PTGFRN 1 1 1 3

hsa-miR-17-5p ARHGAP1 1 1 1 3

hsa-miR-301b-3p EGLN3 1 1 1 3

hsa-miR-17-5p PTPN4 1 1 1 3

hsa-miR-301b-3p RLIM 1 1 1 3

hsa-miR-20b-5p FBXL5 1 1 1 3

hsa-miR-24-3p BCL2L11 1 1 1 3

hsa-miR-24-3p ABHD2 1 1 1 3

hsa-miR-125b-5p KLF13 1 1 1 3

hsa-miR-20b-5p GNB5 1 1 1 3

hsa-miR-17-5p CENPQ 1 1 1 3

hsa-miR-17-5p ATAD2 1 1 1 3

hsa-miR-363-3p MYO1B 1 1 1 3

hsa-miR-20b-5p BBX 1 1 1 3

hsa-miR-216b-5p C11orf57 1 1 1 3

hsa-miR-17-5p CIT 1 1 1 3

hsa-miR-107 FGFRL1 1 1 1 3

hsa-miR-507 LIMS1 1 1 1 3

hsa-miR-338-3p ZDHHC18 1 1 1 3

hsa-miR-20b-5p ENPP5 1 1 1 3

hsa-miR-301b-3p MAP3K9 1 1 1 3

hsa-miR-17-5p CPOX 1 1 1 3

hsa-miR-20b-5p DYNC1LI2 1 1 1 3

hsa-miR-129-5p ABCC5 1 1 1 3

hsa-miR-301b-3p NUS1 1 1 1 3

hsa-miR-1297 CTNS 1 1 1 3

hsa-miR-129-5p ADD3 1 1 1 3

hsa-miR-20b-5p MAPRE3 1 1 1 3

hsa-miR-24-3p AP5M1 1 1 1 3

hsa-miR-17-5p POLQ 1 1 1 3

hsa-miR-17-5p GNB5 1 1 1 3

hsa-miR-129-5p TERF2 1 1 1 3

hsa-miR-125b-5p VPS4B 1 1 1 3

hsa-miR-761 ERC1 1 1 1 3

hsa-miR-17-5p CHIC1 1 1 1 3

hsa-miR-193a-3p MSANTD2 1 1 1 3

hsa-miR-17-5p GPAM 1 1 1 3

hsa-miR-27a-3p RNF139 1 1 1 3

hsa-miR-27a-3p RYBP 1 1 1 3

hsa-miR-125b-5p DHX33 1 1 1 3

hsa-miR-20b-5p HSPA8 1 1 1 3

hsa-miR-1244 SMAD7 1 1 1 3

hsa-miR-107 FAM103A1 1 1 1 3

hsa-miR-363-3p KIAA1109 1 1 1 3

hsa-miR-17-5p HAS2 1 1 1 3

hsa-miR-24-3p RNF2 1 1 1 3

hsa-miR-17-5p C14orf28 1 1 1 3

hsa-miR-301b-3p BTG1 1 1 1 3

hsa-miR-125a-5p TRPS1 1 1 1 3

hsa-miR-301b-3p ACBD5 1 1 1 3

hsa-miR-20b-5p TXNIP 1 1 1 3

hsa-miR-216b-5p SMAD1 1 1 1 3

hsa-miR-613 RNF138 1 1 1 3

hsa-miR-193a-3p DYRK2 1 1 1 3

hsa-miR-17-5p SNTB2 1 1 1 3

hsa-miR-125b-5p EDEM1 1 1 1 3

hsa-miR-17-5p RRAGD 1 1 1 3

hsa-miR-363-3p YIPF4 1 1 1 3

hsa-miR-20b-5p RPA2 1 1 1 3

hsa-miR-27a-3p C6orf120 1 1 1 3

hsa-miR-10a-5p NR2C2 1 1 1 3

hsa-miR-125a-5p EIF4EBP1 1 1 1 3

hsa-miR-27a-3p UBR5 1 1 1 3

hsa-miR-338-3p PREX2 1 1 1 3

hsa-miR-507 IMPAD1 1 1 1 3

hsa-miR-22-3p PDIK1L 1 1 1 3

hsa-miR-107 ATP13A3 1 1 1 3

hsa-miR-17-5p KIAA1147 1 1 1 3

hsa-miR-363-3p CNIH1 1 1 1 3

hsa-miR-301b-3p ZFYVE26 1 1 1 3

hsa-miR-129-5p UNC5D 1 1 1 3

hsa-miR-20b-5p PRR14L 1 1 1 3

hsa-miR-206 TKT 1 1 1 3

hsa-miR-363-3p SOX11 1 1 1 3

hsa-miR-20b-5p ZNF532 1 1 1 3

hsa-miR-24-3p CRIPT 1 1 1 3

hsa-miR-129-5p GABBR2 1 1 1 3

hsa-miR-206 STC2 1 1 1 3

hsa-miR-20b-5p BAMBI 1 1 1 3

hsa-miR-27a-3p PPARG 1 1 1 3

hsa-miR-17-5p MAP3K8 1 1 1 3

hsa-miR-129-5p DAB2 1 1 1 3

hsa-miR-17-5p KLHL28 1 1 1 3

hsa-miR-17-5p SPRED1 1 1 1 3

hsa-miR-20b-5p YOD1 1 1 1 3

hsa-miR-20b-5p CCSER2 1 1 1 3

hsa-miR-27a-3p KIAA1551 1 1 1 3

hsa-miR-125b-5p ZSWIM6 1 1 1 3

hsa-miR-17-5p MMP2 1 1 1 3

hsa-miR-301b-3p PPP6R3 1 1 1 3

hsa-miR-1297 ZDHHC18 1 1 1 3

hsa-miR-301b-3p ZNF800 1 1 1 3

hsa-miR-107 TWF1 1 1 1 3

hsa-miR-206 KRAS 1 1 1 3

hsa-miR-129-5p REEP1 1 1 1 3

hsa-miR-125b-5p ENPEP 1 1 1 3

hsa-miR-140-5p YOD1 1 1 1 3

hsa-miR-129-5p SORBS2 1 1 1 3

hsa-miR-1297 RNF6 1 1 1 3

hsa-miR-301b-3p MB21D2 1 1 1 3

hsa-miR-129-5p SBNO1 1 1 1 3

hsa-miR-17-5p KAT2B 1 1 1 3

hsa-miR-17-5p PANK3 1 1 1 3

hsa-miR-301b-3p MCC 1 1 1 3

hsa-miR-17-5p BTG3 1 1 1 3

hsa-miR-27a-3p NF1 1 1 1 3

hsa-miR-1297 PMAIP1 1 1 1 3

hsa-miR-20b-5p CRY2 1 1 1 3

hsa-miR-3619-5p TFAP2C 1 1 1 3

hsa-miR-27a-3p ZFP36L2 1 1 1 3

hsa-miR-20b-5p HMBOX1 1 1 1 3

hsa-miR-24-3p ZNF217 1 1 1 3

hsa-miR-140-5p TSPAN12 1 1 1 3

hsa-miR-17-5p RCCD1 1 1 1 3

hsa-miR-507 GTF2H1 1 1 1 3

hsa-miR-17-5p CLOCK 1 1 1 3

hsa-miR-17-5p LASP1 1 1 1 3

hsa-miR-140-5p FGF9 1 1 1 3

hsa-miR-17-5p NCOA3 1 1 1 3

hsa-miR-23b-3p ANKRD17 1 1 1 3

hsa-miR-425-5p RAB31 1 1 1 3

hsa-miR-425-5p SPPL2A 1 1 1 3

hsa-miR-20b-5p SGTB 1 1 1 3

hsa-miR-125a-5p TEF 1 1 1 3

hsa-miR-301b-3p MID1IP1 1 1 1 3

hsa-miR-20b-5p DNAJB9 1 1 1 3

hsa-miR-23b-3p FUT4 1 1 1 3

hsa-miR-125a-5p REST 1 1 1 3

hsa-miR-301b-3p KIF13A 1 1 1 3

hsa-miR-17-5p ZBTB9 1 1 1 3

hsa-miR-363-3p ITPR1 1 1 1 3

hsa-miR-20b-5p PKNOX1 1 1 1 3

hsa-miR-17-5p NRBP1 1 1 1 3

hsa-miR-17-5p NFAT5 1 1 1 3

hsa-miR-17-5p RGMB 1 1 1 3

hsa-miR-17-5p GBF1 1 1 1 3

hsa-miR-27a-3p WEE1 1 1 1 3

hsa-miR-3619-5p ZNRF1 1 1 1 3

hsa-miR-125b-5p VDR 1 1 1 3

hsa-miR-20b-5p BMPR2 1 1 1 3

hsa-miR-23b-3p ZMYM2 1 1 1 3

hsa-miR-17-5p TMEM123 1 1 1 3

hsa-miR-20b-5p MASTL 1 1 1 3

hsa-miR-17-5p DYNC1LI2 1 1 1 3

hsa-miR-27a-3p GNG12 1 1 1 3

hsa-miR-20b-5p FJX1 1 1 1 3

hsa-miR-1297 GSK3B 1 1 1 3

hsa-miR-193a-3p SLC10A6 1 1 1 3

hsa-miR-23b-3p HMGB2 1 1 1 3

hsa-miR-20b-5p U2SURP 1 1 1 3

hsa-miR-24-3p KCNK2 1 1 1 3

hsa-miR-20b-5p CRK 1 1 1 3

hsa-miR-761 TBPL1 1 1 1 3

hsa-miR-107 GABRB1 1 1 1 3

hsa-miR-125b-5p SLC7A1 1 1 1 3

hsa-miR-20b-5p PIP4K2C 1 1 1 3

hsa-miR-3619-5p TRAF1 1 1 1 3

hsa-miR-301b-3p PTPN4 1 1 1 3

hsa-miR-24-3p TMEM173 1 1 1 3

hsa-miR-107 TRIM35 1 1 1 3

hsa-miR-20b-5p HAUS8 1 1 1 3

hsa-miR-1244 SAT1 1 1 1 3

hsa-miR-129-5p ZNF25 1 1 1 3

hsa-miR-338-3p SGTB 1 1 1 3

hsa-miR-17-5p CMPK1 1 1 1 3

hsa-miR-23b-3p CHUK 1 1 1 3

hsa-miR-301b-3p IRF1 1 1 1 3

hsa-miR-17-5p EEA1 1 1 1 3

hsa-miR-24-3p KLHDC3 1 1 1 3

hsa-miR-17-5p RUNDC1 1 1 1 3

hsa-miR-20b-5p OXR1 1 1 1 3

hsa-miR-301b-3p GRB10 1 1 1 3

hsa-miR-125a-5p ANAPC16 1 1 1 3

hsa-miR-17-5p DCBLD2 1 1 1 3

hsa-miR-125b-5p IRF4 1 1 1 3

hsa-miR-20b-5p CADM2 1 1 1 3

hsa-miR-338-3p PFAS 1 1 1 3

hsa-miR-20b-5p SLAIN2 1 1 1 3

hsa-miR-20b-5p KMT2B 1 1 1 3

hsa-miR-301b-3p CLIP1 1 1 1 3

hsa-miR-301b-3p TNRC6A 1 1 1 3

hsa-miR-20b-5p MKNK2 1 1 1 3

hsa-miR-17-5p HIF1A 1 1 1 3

hsa-miR-17-5p PLS1 1 1 1 3

hsa-miR-27a-3p GRB2 1 1 1 3

hsa-miR-301b-3p CLCN3 1 1 1 3

hsa-miR-216b-5p ZNF566 1 1 1 3

hsa-miR-20b-5p TET3 1 1 1 3

hsa-miR-17-5p TET3 1 1 1 3

hsa-miR-507 AKAP1 1 1 1 3

hsa-miR-125b-5p NCOR2 1 1 1 3

hsa-miR-107 USP42 1 1 1 3

hsa-miR-125a-5p ZSWIM6 1 1 1 3

hsa-miR-206 PGD 1 1 1 3

hsa-miR-22-3p RAB5B 1 1 1 3

hsa-miR-140-5p MMD 1 1 1 3

hsa-miR-20b-5p SSX2IP 1 1 1 3

hsa-miR-20b-5p TNFRSF21 1 1 1 3

hsa-miR-20b-5p MAP3K2 1 1 1 3

hsa-miR-107 FURIN 1 1 1 3

hsa-miR-17-5p ACSL4 1 1 1 3

hsa-miR-338-3p NOVA1 1 1 1 3

hsa-miR-27a-3p EIF5 1 1 1 3

hsa-miR-206 FRS2 1 1 1 3

hsa-miR-23b-3p STAT5B 1 1 1 3

hsa-miR-490-3p NUFIP2 1 1 1 3

hsa-miR-17-5p 2-Sep 1 1 1 3

hsa-miR-20b-5p DENND5B 1 1 1 3

hsa-miR-425-5p MAP3K5 1 1 1 3

hsa-miR-27a-3p LPCAT1 1 1 1 3

hsa-miR-20b-5p ANKRD50 1 1 1 3

hsa-miR-761 NAP1L4 1 1 1 3

hsa-miR-20b-5p LYSMD3 1 1 1 3

hsa-miR-301b-3p DEPDC1 1 1 1 3

hsa-miR-363-3p SLC25A36 1 1 1 3

hsa-miR-24-3p SPIN4 1 1 1 3

hsa-miR-20b-5p POLQ 1 1 1 3

hsa-miR-1297 STRADB 1 1 1 3

hsa-miR-613 MATR3 1 1 1 3

hsa-miR-27a-3p GPAM 1 1 1 3

hsa-miR-20b-5p SNTB2 1 1 1 3

hsa-miR-17-5p RHOC 1 1 1 3

hsa-miR-20b-5p ATG14 1 1 1 3

hsa-miR-17-5p SHOC2 1 1 1 3

hsa-miR-363-3p SLC25A32 1 1 1 3

hsa-miR-363-3p SLX4 1 1 1 3

hsa-miR-338-3p MRPS23 1 1 1 3

hsa-miR-125b-5p EIF4EBP1 1 1 1 3

hsa-miR-17-5p STX6 1 1 1 3

hsa-miR-107 RAB1B 1 1 1 3

hsa-miR-1297 CCND2 1 1 1 3

hsa-miR-125b-5p STAT3 1 1 1 3

hsa-miR-1297 FAM177A1 1 1 1 3

hsa-miR-107 SREK1 1 1 1 3

hsa-miR-363-3p GOLGA8A 1 1 1 3

hsa-miR-27a-3p SEMA6A 1 1 1 3

hsa-miR-206 EIF1AX 1 1 1 3

hsa-miR-363-3p PAX9 1 1 1 3

hsa-miR-10a-5p CADM1 1 1 1 3

hsa-miR-27a-3p MYT1 1 1 1 3

hsa-miR-125b-5p MCL1 1 1 1 3

hsa-miR-1297 ZBTB18 1 1 1 3

hsa-miR-17-5p ANKRD12 1 1 1 3

hsa-miR-613 LRRC59 1 1 1 3

hsa-miR-363-3p PCMTD1 1 1 1 3

hsa-miR-1297 TRIB2 1 1 1 3

hsa-miR-27a-3p E2F7 1 1 1 3

hsa-miR-1297 ACBD5 1 1 1 3

hsa-miR-10a-5p MAP3K7 1 1 1 3

hsa-miR-17-5p ATG14 1 1 1 3

hsa-miR-20b-5p ABCA1 1 1 1 3

hsa-miR-363-3p CNNM4 1 1 1 3

hsa-miR-107 CDADC1 1 1 1 3

hsa-miR-1297 ZNF410 1 1 1 3

hsa-miR-24-3p MARCKSL1 1 1 1 3

hsa-miR-876-3p RBBP6 1 1 1 3

hsa-miR-17-5p RPA2 1 1 1 3

hsa-miR-17-5p MAPK9 1 1 1 3

hsa-miR-125b-5p ANAPC16 1 1 1 3

hsa-miR-125a-5p IRF4 1 1 1 3

hsa-miR-363-3p PPP1R37 1 1 1 3

hsa-miR-24-3p HIC2 1 1 1 3

hsa-miR-27a-3p TAOK1 1 1 1 3

hsa-miR-20b-5p OCRL 1 1 1 3

hsa-miR-17-5p ETV1 1 1 1 3

hsa-miR-23b-3p ZCCHC2 1 1 1 3

hsa-miR-20b-5p NHLRC3 1 1 1 3

hsa-miR-24-3p LIMD1 1 1 1 3

hsa-miR-20b-5p CLIP4 1 1 1 3

hsa-miR-363-3p UBXN4 1 1 1 3

hsa-miR-27a-3p SPRY2 1 1 1 3

hsa-miR-1297 FAXC 1 1 1 3

hsa-miR-129-5p PRDM1 1 1 1 3

hsa-miR-20b-5p CNOT6L 1 1 1 3

hsa-miR-17-5p PARD6B 1 1 1 3

hsa-miR-17-5p EZH1 1 1 1 3

hsa-miR-20b-5p RAPGEF4 1 1 1 3

hsa-miR-27a-3p LONRF1 1 1 1 3

hsa-miR-20b-5p PHF6 1 1 1 3

hsa-miR-27a-3p TGFBR3 1 1 1 3

hsa-miR-363-3p TWF1 1 1 1 3

hsa-miR-125b-5p REST 1 1 1 3

hsa-miR-1244 ITM2A 1 1 1 3

hsa-miR-1297 MTDH 1 1 1 3

hsa-miR-20b-5p RHOC 1 1 1 3

hsa-miR-20b-5p ATG16L1 1 1 1 3

hsa-miR-17-5p MASTL 1 1 1 3

hsa-miR-17-5p ZFYVE9 1 1 1 3

hsa-miR-125a-5p OSBPL9 1 1 1 3

hsa-miR-425-5p NRAS 1 1 1 3

hsa-miR-301b-3p CAPRIN2 1 1 1 3

hsa-miR-17-5p TNFAIP1 1 1 1 3

hsa-miR-425-5p BCOR 1 1 1 3

hsa-miR-20b-5p CREB1 1 1 1 3

hsa-miR-10a-5p CSRNP3 1 1 1 3

hsa-miR-363-3p BTG2 1 1 1 3

hsa-miR-301b-3p NAA50 1 1 1 3

hsa-miR-10a-5p LHFPL4 1 1 1 3

hsa-miR-20b-5p FNBP1L 1 1 1 3

hsa-miR-107 AMOT 1 1 1 3

hsa-miR-425-5p RNF168 1 1 1 3

hsa-miR-27a-3p C8orf4 1 1 1 3

hsa-miR-761 C10orf76 1 1 1 3

hsa-miR-363-3p RNF4 1 1 1 3

hsa-miR-301b-3p LDLR 1 1 1 3

hsa-miR-27a-3p PSPC1 1 1 1 3

hsa-miR-125b-5p TEF 1 1 1 3

hsa-miR-17-5p RUNX3 1 1 1 3

hsa-miR-363-3p NUFIP2 1 1 1 3
